# Ginsenoside Rb1 prevents MPTP-induced changes in hippocampal memory via regulation of the α-synuclein/PSD-95 pathway

**DOI:** 10.18632/aging.101884

**Published:** 2019-04-04

**Authors:** Shaogang Qu, Xingjun Meng, Yan Liu, Xiuping Zhang, Yunlong Zhang

**Affiliations:** 1 Central Laboratory and Department of Neurology, Shunde Hospital, Southern Medical University (The First People’s Hospital of Shunde Foshan), Foshan 528300, China; 2 Department of Traditional Chinese Medicine, Medical College, Xiamen University, Xiamen 361102, China; 3 Teaching Center of Experimental Medicine, School of Basic Medical Sciences, Southern Medical University, Guangzhou 510515, China; 4 Key Laboratory of Neuroscience, School of Basic Medical Sciences, Guangzhou Medical University, Guangzhou 511436, China; 5 Shenzhen Research Institute of Xiamen University, Shenzhen 518000, China

**Keywords:** Parkinson’s disease, ginsenoside Rb1, memory deficits, α-synuclein, synaptic plasticity

## Abstract

Memory deficiency is a common non-motor symptom of Parkinson’s disease (PD), and conventionally, α-synuclein is considered to be an important biomarker for both motor and cognitive characteristics attributed to PD. However, the role of physiological α-synuclein in cognitive impairment remains undetermined. Ginsenoside Rb1 has been shown to protect dopaminergic neurons (DA) from death and inhibit α-synuclein fibrillation and toxicity *in vitro*. Our recent study also revealed that ginsenoside Rb1 ameliorates motor deficits and prevents DA neuron death via upregulating glutamate transporter GLT-1 in the 1-methyl-4-phenyl-1,2,3,6-tetrahydropyridine (MPTP) mouse model of PD. Whether Rb1 can improve memory deficiency and the underlying mechanism is still unknown. In this study, we found that Rb1 can prevent the spatial learning and memory deficits, increase long-term potentiation (LTP) and hippocampal glutamatergic transmission in the MPTP mouse model. The underlying neuroprotective mechanism of Rb1-improved synaptic plasticity involves Rb1 promoting hippocampal CA3 α-synuclein expression, restoring the glutamate in the CA3-schaffer collateral-CA1 pathway, and sequentially increasing postsynaptic density-95 (PSD-95) expression. Thus, we provide evidence that Rb1 modulates memory function, synaptic plasticity, and excitatory transmission via the trans-synaptic α-synuclein/PSD-95 pathway. Our findings suggest that Rb1 may serve as a functional drug in treating the memory deficiency in PD.

## INTRODUCTION

Parkinson’s disease (PD) is the second most common neurodegenerative disorder, characterized by movement disorders like akinesia, rigidity, resting tremor, and postural instability. Recently, the non-motor symptoms of PD, such as cognitive, sensory, psychiatric, and autonomic dysfunction, have drawn increasing attention [1]. In particular, cognitive dysfunction is one of the most prevalent non-motor symptoms in PD, affecting memory, attention, and executive as well as visual-spatial abilities [2, 3]. Almost 20–33% of patients already experience mild cognitive impairment at the time of PD diagnosis (called PD-MCI) [4, 5]. Without prior to the clinical suspicion of cognitive impairment, 34% of early-stage PD patients show the cognitive impairment, and this cognitive impairment is associated with motor symptoms severity, such as bradykinesia, rigidity and axial symptoms [6]. Aside from the 20–25% of patients with Parkinson’s disease who have mild cognitive impairment, 30% are reported to have dementia [2]. It has been noted that the incidence of dementia is nearly 100 per 100,000 patient-years in prevalence samples of PD [7]. In addition, the bilateral insula and right hippocampus were identified as regions of structural atrophy in PD patients with dementia (PDD) [8].

Dopaminergic (DA) neuron degeneration in the substantia nigra and striatum, together with the formation of Lewy bodies mediated by α-synuclein aggregation, are the hallmark of PD pathology [9–12]. Both total plasma and nervous system-derived exosomal and cerebrospinal fluid α-synuclein concentrations contribute to the cognitive impairment in early stages of PD progression [13, 14]. Genetically, *SNCA* (α-synuclein gene) duplications, *SNCA* Rep1 microsatellites, and *SNCA* polymorphism contribute to the cognitive impairments and dementia in PD [15–20], and *DYRK1A* polymorphisms, which encode a kinase that phosphorylates α-synuclein, are associated with dementia in PD [21]. A great deal of evidence suggests that therapies aimed at α-synuclein suppression may be beneficial for the cognitive impairment in PD [22–26]. However, α-synuclein also plays important roles in the release of synaptic vesicles and synaptic membrane recycling in normal neurons. Physiologically, α-synuclein is localized at presynaptic terminals throughout the mammalian brain and is involved in the generation and maintenance of synapses [27–29]. In the hippocampal synapse, α-synuclein expression is highly concentrated in the granular and polymorphic layers of the dentate gyrus (DG) as well as in the CA2 and CA3 fields [30]. In particular, α-synuclein is found to be localized at excitatory presynapses and co-expressed with vesicular glutamate transporter1 (vGluT1), an excitatory presynaptic marker [31, 32]. Moreover, α-synuclein knockout mice show reduced learning ability in working and spatial memory tests [33], suggesting that α-synuclein may play an important role in learning and memory. However, the function and mechanism of hippocampal α-synuclein in the cognitive impairment in PD are still not fully understood.

Ginsenoside Rb1, the primary active ingredient of *Panax ginseng*, has been reported to be neuroprotective in PD models via protecting DA neurons [34]. Our recent work showed that Rb1 can ameliorate motor deficits in PD animal models through protecting DA neuron from glutamate transporter GLT-1 reduction-mediated glutamate excitotoxicity [35]. Previously, Rb1 was reported to regulate hippocampal neurogenesis, stress-induced brain-derived neurotrophic factor (BDNF) changes, and hippocampal synaptic density to improve spatial learning and memory [36–38]. Whether Rb1 can improve the cognitive impairment in PD remains unknown.

In the present study, we address the role of Rb1 in the cognitive impairment in PD, involving spatial learning and memory. We demonstrate that Rb1 improves the spatial learning and memory deficits via the trans-synaptic hippocampal α-synuclein/postsynaptic density protein-95 (PSD-95) pathway. The findings serve as a theoretical basis for the exploration of Rb1 as a functional drug for PD patients with cognitive impairment.

## RESULTS

### Rb1 prevents memory deficits in MPTP-treated mice

**Figure 1:**
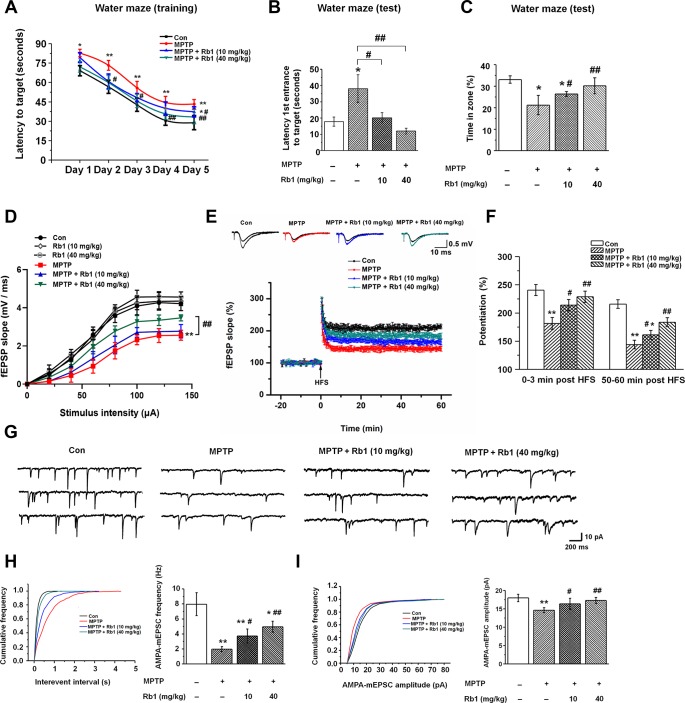
**Rb1 prevents cognitive impairment and dysfunctional glutamatergic transmission in the MPTP mouse model of PD.** (**A**–**C**) Morris water maze tests were conducted after treatment with MPTP or different doses of Rb1. Mice were analyzed for (A) the escape latency during a 5-day training course. In the probe tests, mice were analyzed (B) for the escape latency, and (C) the time spent in the target zone. n = 12 per group. (**D**) Input–output relations generated by stimulating the SCs and recording in CA1 stratum radiatum. n = 6–10. (**E**) The effect of Rb1 on the LTP at the SC-CA1 synapses was recorded in MPTP-treated mice. The middle image shows representative traces of fEPSP recordings of responses before and 50 min after high-frequency stimulation (HFS; arrow). (**F**) Quantitative analysis of data in e. The level of fEPSP potentiation was determined at a mean of 0–3 min and 50–60 min after high-frequency stimulation. n = 5–8. (**G**) Representative traces of APMA receptor-mediated mEPSCs. All mEPSCs were recorded at a holding potential of −65 mV. (**H**) Cumulative frequency plots of the inter-event interval (left) and quantitative analysis of the frequency of APMA receptor-mediated mEPSCs (right). (**I**) Cumulative frequency plots of the amplitude (left) and quantitative analysis of the amplitude of APMA receptor-mediated mEPSCs (right). n = 11–15 per group. Data were obtained from the whole-cell recordings of the pyramidal neurons in the hippocampal CA3 region from the four groups of mice. Results are expressed as the mean ± SEM. ^**^p < 0.01, ^*^p < 0.05 vs. control group; ^##^*p* < 0.01, ^#^*p* < 0.05 vs. MPTP group. Statistical significance was determined by one-way ANOVA and Bonferroni tests as *post hoc* comparisons.

Neurotoxins, including MPTP, 6-hydroxydopamine (6-OHDA), and rotenone, are the classic drugs used to generate PD animal models. These neurotoxins not only impair motor functions, but also affect cognitive networks [39, 40], and we used MPTP mice model in this study. The timeline of the experiments was shown in Supplementary Figure 1. Firstly, we performed pole-climbing test, grasping test and rotarod test to confirm the effects of Rb1 on the motor dysfunction in the MPTP mice model, 10 mg/kg and 40 mg/kg Rb1 improved the movement disorder in MPTP mice model (Supplementary Figure 2A–2C), which is consistent with our recent study [35]. Since MPTP has been proposed to induce hippocampal memory deficits and participate in the pathophysiology of the non-motor symptoms of PD [39, 41], then in this study, we treated MPTP-lesioned mice with Rb1 and subjected them to the Morris water maze to assess their spatial learning and memory. In this test, mice were dropped into a pool and allowed to swim to locate a submerged platform to escape swimming. MPTP-lesioned mice showed impaired learning response with increased escape latency during the five-day training phase; however, low-dose (10 mg/kg) and high-dose (40 mg/kg) Rb1 treatment decreased escape latency to the target (Figure 1A). Moreover, in the probe trial test, in which the hidden platform was removed on Day 6, mice that received low-dose (10 mg/kg) or high-dose (40 mg/kg) Rb1 took less time to reach the target (F _3, 44_ = 10.445, *P* < 0.001, post-hoc *P* = 0.023 for 10 mg/kg Rb1 and post-hoc *P* < 0.001 for 40 mg/kg Rb1; Figure 1B) and spent significantly more time in the target quadrant (F _3, 44_ = 14.388, *P* < 0.001, post-hoc *P* = 0.026 for 10 mg/kg Rb1 and post-hoc *P* < 0.001 for 40 mg/kg Rb1; Figure 1C) compared with MPTP-treated mice, and these mice showed no significant difference in the target crossing in the water maze (F _3, 44_ = 0.009, *P* = 0.999, no significance for 10 mg/kg Rb1 and 40 mg/kg Rb1; Supplementary Figure 2D). The swim speeds of these mice in four groups showed no obvious difference, suggesting the motor dysfunction may not affect mice performance in the water maze test (Supplementary Figure 2E and 2F). These results indicate that Rb1 can improve the spatial learning and memory deficits in MPTP-lesioned mice.

### Rb1 prevents MPTP-impaired hippocampal synaptic plasticity, glutamatergic transmission, and neuronal activity

Since synaptic plasticity and transmission are responsible for the formation of memory [42, 43], and in order to eliminate the possible effects of motor dysfunction on the Morris water maze test, we next examined the effects of Rb1 on the long-term potentiation (LTP) and excitatory synaptic transmission in the hippocampus in the MPTP-treated mice. We first detected the synaptic function in the Schaffer collateral pathway (SC-CA1) in hippocampal slices, and fEPSPs were recorded in the CA1 stratum radiatum by stimulating the SC/commissural pathway at various intensities. No significant difference in fEPSP slopes was detected at the tested stimulus intensities in the CA1 area of Con, low-dose (10 mg/kg) and high-dose (40 mg/kg) Rb1 (Figure 1D), suggesting similar strength of basal synaptic transmission. However, high-dose (40 mg/kg) Rb1 increased the fEPSP slopes in CA1 hippocampal slices compared with those of MPTP or MPTP + Rb1 (10 mg/kg) group (Figure 1D), indicating that Rb1 improved the synaptic transmission in MPTP mice model. Next, we determined the LTP at the SC-CA1 synapses, both the post-tetanic potentiation and LTP at SC-CA1 synapses were reduced in hippocampal slices from the mice with MPTP treatment (Figure 1E and 1F), consistent with our previous findings [44]. Our data showed that compared with the control, treatment with MPTP significantly decreased the LTP amplitude at 0–3 min and 50–60 min after LTP induction (F _3, 22_ = 21.254, *P* < 0.001, post-hoc *P* < 0.001 for 0-3 min; F _3, 22_ = 53.715, *P* < 0.001, post-hoc *P* < 0.001 for 50-60 min Figure 1F), which was prevented by both low-dose (10 mg/kg) and high-dose (40 mg/kg) Rb1 treatment (F _3, 22_ = 21.254, *P* < 0.001, post-hoc *P* = 0.032 for 10 mg/kg Rb1 and post-hoc *P* < 0.001 for 40 mg/kg Rb1 at 0-3 min; F _3, 22_ = 53.715, *P* < 0.001, post-hoc *P* = 0.037 for 10 mg/kg Rb1 and post-hoc *P* = 0.002 for 40 mg/kg Rb1 at 50-60 min; Figure 1F). These data suggest that Rb1 prevents the hippocampal synaptic plasticity deficit in the MPTP model.

Schaffer collateral inputs from CA3 to CA1 is responsible for the formation of LTP, and then we examined the excitatory synaptic transmission in hippocampal CA3 pyramidal neurons. α-Amino-3-hydroxy-5-methyl-4-isoxazolepropionic acid (AMPA) receptor-mediated miniature excitatory postsynaptic currents (mEPSCs) were recorded in whole-cell configuration. Both the amplitudes and frequencies of AMPA receptor-mediated mEPSCs were significantly reduced in the CA3 pyramidal neurons in MPTP-treated mice compared with the control (Figure 1G–1I). However, both low-dose (10 mg/kg) and-high dose (40 mg/kg) Rb1 treatment increased the frequency and amplitudes of AMPA receptor-mediated mEPSCs in MPTP-treated hippocampal CA3 pyramidal neurons (frequency: F _3, 46_ = 82.641, *P* < 0.001, post-hoc *P* = 0.011 for 10 mg/kg Rb1 and post-hoc *P* < 0.001 for 40 mg/kg Rb1; Figure 1H; amplitudes: F _3, 46_ = 17.513, *P* < 0.001, post-hoc *P* = 0.031 for 10 mg/kg Rb1 and *P* < 0.001 for 40 mg/kg Rb1, respectively; Figure 1I). These results suggest that Rb1 prevents the deficits of glutamatergic synaptic transmission at the CA3 synapses.

**Figure 2:**
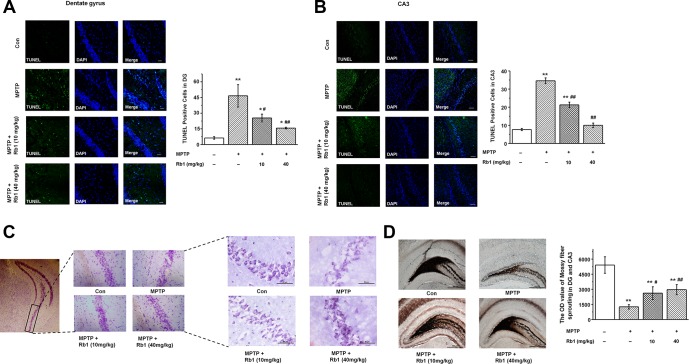
**Rb1 protects hippocampal neurons from death in MPTP-treated mice.** (**A** and **B**) TUNEL staining showed the apoptosis of hippocampal neurons in the DG and CA3 regions. Scale bar, 30 μm in (A) and 60 μm in (B). (**C**) Nissl staining of hippocampal neurons in the CA3 regions. Scale bar, 100 μm for the low-power field in the left and 50 μm for the high-power field in the right. (**D**) Timm staining of mossy fiber in the DG and CA3 regions. Scale bar, 500 μm. n = 10 per group. Results are expressed as the mean ± SEM. ^**^p < 0.01, ^*^p < 0.05 vs. control group; ^##^*p* < 0.01, ^#^*p* < 0.05 vs. MPTP group. Statistical significance was determined by one-way ANOVA and Bonferroni tests as *post hoc* comparisons.

Having demonstrated that Rb1 improves MPTP-induced spatial learning and memory deficits involved in regulating synaptic plasticity and glutamatergic synaptic transmission at the CA3 synapses, we then wanted to know whether Rb1 protects pyramidal neurons from MPTP-induced death in the CA3 and DG. TUNEL staining showed that MPTP induced pyramidal neuron death in the DG and CA3, and both low-dose (10 mg/kg) and high-dose (40 mg/kg) Rb1 treatment prevented the neuronal death (F _3, 36_ = 78.777, *P* < 0.001, post-hoc *P* = 0.044 for 10 mg/kg Rb1 and post-hoc *P* < 0.001 for 40 mg/kg Rb1; Figure 2A; F _3, 36_ = 74.082, *P* < 0.001, post-hoc *P* < 0.001 for 10 mg/kg Rb1 and 40 mg/kg Rb1, respectively; Figure 2B). Moreover, Nissl staining showed that intraneural Nissl bodies were lightly stained and appeared to be sparsely arranged in MPTP-treated mice, and deeper-stained Nissl bodies with higher density in hippocampal neurons were found in the Rb1 treatment group (Figure 2C). Since mossy fiber-CA3 synapses mediate homeostatic plasticity in mature hippocampal neurons [45], MPTP induced dysfunctional CA3 synaptic transmission and neuron loss. We then used Timm staining to examine the effect of Rb1 on the mossy fibers in DG and CA3. Here, the Timm staining showed that mossy fiber intensity was significantly decreased in DG and CA3 in MPTP-treated mice, and Rb1 prevented the reduced mossy fiber intensity (F _3, 36_ = 25.690, *P* < 0.001, post-hoc *P* = 0.045 for 10 mg/kg Rb1 and post-hoc *P* = 0.004 for 40 mg/kg Rb1; Figure 2D). These results support the neuroprotective effects of Rb1 in MPTP-mediated hippocampal synaptic plasticity.

### Rb1 prevented the MPTP-induced decrease of α-synuclein expression in the hippocampal CA3 region

We found that Rb1 prevented MPTP-impaired hippocampal synaptic plasticity and AMPA-mediated glutamatergic transmission, we then examined the effects of Rb1 on the expression of glutamate receptors, glutamate transporters, and pre- and post-synaptic proteins. We found that at the total protein level, Rb1 showed no obvious effects on the subunits of N-methyl-D-aspartate (NMDA) receptors (NMDAR1, NMDAR2B), AMPA receptors (GluA1, GluA2), glutamate transporters (GLT-1, GLAST), or presynaptic proteins (synapsin, synaptophysin, syntaxin) in the hippocampus in MPTP-treated mice (Supplementary Figure 3A–3C). However, Rb1 significantly prevented MPTP-reduced PSD-95 expression, which is an excitatory postsynaptic localization protein and is required for activity-dependent synapse stabilization [46] (F _3, 20_ = 35.187, *P* < 0.001, post-hoc *P* < 0.001 for 10 mg/kg Rb1 and 40 mg/kg Rb1, respectively; Supplementary Figure 3C). Meanwhile, at the membrane protein level, only a high dose (40 mg/kg) of Rb1 partially prevented GluA1 expression (F _3, 20_ = 8.438, *P* = 0.001, post-hoc *P* = 0.224 for 10 mg/kg Rb1 and post-hoc *P* = 0.001 for 40 mg/kg Rb1; Supplementary Figure 3E), and Rb1 showed no obvious effects on the other NMDA and AMPA receptors’ expression (Supplementary Figure 3D and 3E).

**Figure 3:**
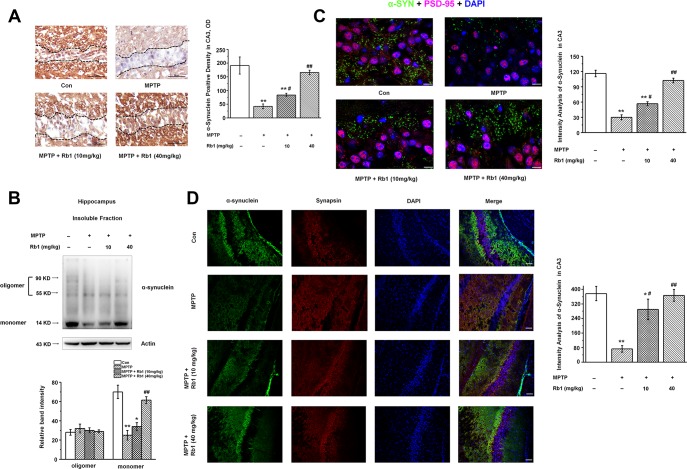
**Rb1 increases α-synuclein expression in the hippocampus in the MPTP-treated mice.** (**A**) Immunohistochemical staining of α-synuclein in the CA3 region. Note that MPTP induced the decrease of α-synuclein, which separated by the black dotted box. Scale bar, 50 μm. (**B**) Oligomeric and monomeric α-synuclein was extracted as stated in Material and Methods and examined by Western blotting. (**C** and **D**) Double staining of α-synuclein with PSD-95 (C) or synapsin (D). Scale bar, 10 μm in (C) and 60 μm in (D). n = 7 per group. Western blotting results are from two of the six mice in each group and are expressed as the mean ± SEM of three experiments. ^**^p < 0.01, ^*^p < 0.05 vs. control group; ^##^*p* < 0.01, ^#^*p* < 0.05 vs. MPTP group. Statistical significance was determined by one-way ANOVA and Bonferroni tests as *post hoc* comparisons.

Since α-synuclein is a vertebrate-specific component of presynaptic nerve terminals that may function in modulating synaptic transmission, and the presence of α-synuclein in Lewy bodies is a pathological hallmark in PD, we also examined the α-synuclein expression in the hippocampus. Interestingly, we found that α-synuclein was significantly decreased in the hippocampal CA3 region, and Rb1 increased α-synuclein expression in this region (F _3, 24_ = 19.045, *P* < 0.001, post-hoc *P* = 0.030 for 10 mg/kg Rb1 and post- hoc *P* = 0.007 for 40 mg/kg Rb1; Figure 3A). Moreover, Rb1 increased the monomer rather than oligomer α-synuclein expression in the hippocampus (monomer: F _3, 20_ = 97.453, *P* < 0.001, post-hoc *P* = 0.572 for 10 mg/kg Rb1 and post-hoc *P* = 0.006 for 40 mg/kg Rb1; Figure 3B). Double staining of α-synuclein with PSD-95 or synapsin suggests that α-synuclein expression was also decreased in the CA3 region (Figure 3C and 3D). Meanwhile, both low-dose (10 mg/kg) and high-dose (40 mg/kg) Rb1 treatment prevented MPTP-reduced α-synuclein expression to the normal level in the hippocampal CA3 region (F _3, 24_ = 28.185, *P* < 0.001, post-hoc *P* = 0.020 for 10 mg/kg Rb1 and post-hoc *P* < 0.001 for 40 mg/kg Rb1; Figure 3C; F _3, 24_ = 52.471, *P* < 0.001, post-hoc *P* = 0.015 for 10 mg/kg Rb1 and post-hoc *P* < 0.001 for 40 mg/kg Rb1; Figure 3D). These data indicated that Rb1 increased α-synuclein and PSD-95 expression in the hippocampus in the MPTP-treated mice.

### Knockdown of α-synuclein decreased postsynaptic PSD-95 expression in primary hippocampal neurons *in vitro*

**Figure 4:**
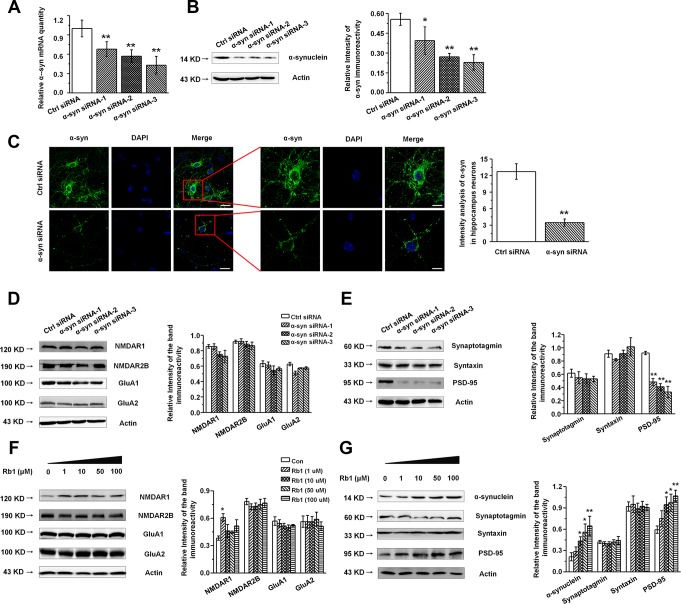
**Knockdown of α-synuclein in the primary cultured hippocampal neuron *in vitro.*** (**A** and **B**) The interference efficiency of α-synuclein siRNA was confirmed by qRT-PCR (A) and Western blotting (B). (**C**) Immunofluorescence staining of control or α-synuclein siRNA in cultured hippocampal neuron. Scale bar, 30 μm for the low-power field in the left and 15 μm for the high-power field in the right. (**D** and **E**) Effects of α-synuclein siRNA on the glutamate receptors and synaptic proteins expressions in cultured hippocampal neuron. (**F**) Effects of Rb1 on the glutamate receptors and synaptic proteins expressions in cultured hippocampal neuron. n= 10 for (C), n = 7 per group for (A), n = 6 for (B) and (D–**G**). Scale bar, 80 μm. Results are expressed as the mean ± SEM of three experiments. ^**^p < 0.01, ^*^p < 0.05 vs. control group. Statistical significance was determined by Student's *t*-test for (C), and one-way ANOVA and Bonferroni tests as *post hoc* comparisons for (A, B) and (D–G).

Our findings indicated that Rb1 increased hippocampal glutamatergic transmission as well as α-synuclein and PSD-95 expression, while Rb1 did not affect the glutamate receptors or other synaptic proteins’ expressions in the hippocampus. Additionally, as α- synuclein were also found to be localized at excitatory presynapses and co-expressed with excitatory presynaptic vGluT-1 [31], we wanted to explore whether Rb1’s modulation of synaptic plasticity and excitatory transmission involved upregulation of α-synuclein expression. We cultured primary hippocampal neurons from C57BL/6 mice E16–18 pups and identified the hippocampal neuron with MAP-2 (Supplementary Figure 4). To knock down expression of α-synuclein in the hippocampal neurons, we generated three siRNA sequences targeting α-synuclein. qPCR and Western blotting indicated that the α-synuclein siRNA interference efficiency was nearly 60% (Figure 4A and 4B). The α-synuclein staining in hippocampal neurons after siRNA treatment is shown in Figure 4C, and siRNA reduced the localization of α-synuclein staining in the cytoplasm and dendrite (Student’s *t*-test, df = 18, *P* < 0.001; Figure 4C). We further examined the effects of α-synuclein knockdown on the expressions of glutamate receptors and synaptic proteins in the primary hippocampal neurons and found that α-synuclein siRNA did not significantly affect NMDA receptor (NMDAR1, NMDAR2B), AMPA receptor (GluA1, GluA2), or presynaptic protein (synaptotagmin, syntaxin) expression (Figure 4D and 4E). Intriguingly, α-synuclein siRNA significantly decreased postsynaptic PSD-95 expression in the hippocampal neurons (F _3, 8_ = 21.297, *P* < 0.001, post-hoc *P* = 0.001 for α-synuclein siRNA-1, and post-hoc *P* < 0.001 for α-synuclein siRNA-2 and 3; Figure 4E), suggesting that α-synuclein may be involved in Rb1’s upregulation of PSD-95 expression in MPTP-treated mice. We then treated the primary hippocampal neurons with different concentrations of Rb1 for 24 h and found that Rb1 significantly increased α-synuclein and PSD-95 expression (α-synuclein: F _4, 27_ = 3.860, *P* = 0.038, post-hoc *P* = 0.282, 0.028, 0.020 and 0.006 for 1, 10, 50 and 100 μM Rb1, respectively; PSD-95: F _4, 27_ = 3.750, *P* = 0.041, post-hoc *P* = 0.586, 0.012, 0.026 and 0.008 for 1, 10, 50 and 100 μM Rb1, respectively; Figure 4G), but it did not affect the NMDA receptors (NMDAR2B), AMPA receptors (GluA1, GluA2), or presynaptic proteins (synaptotagmin, syntaxin) (Figure 4F and 4G). These results indicate that α-synuclein decreased PSD-95 expression in hippocampal neurons.

### α-Synuclein may be involved in Rb1’s regulation of memory function and synaptic plasticity

**Figure 5:**
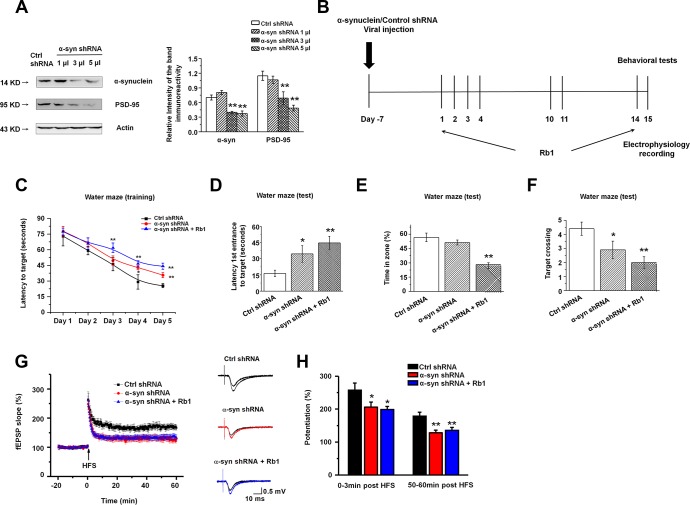
**Knockdown of α-synuclein in hippocampal CA3 impaired learning and memory in normal mice.** (**A**) The interference efficiency of α-synuclein shRNA was confirmed by Western blotting. n = 6 per group. (**B**) Experimental timeline. Seven days after the LV- α-synuclein shRNA or control shRNA virus was stereotaxically injected in the hippocampal CA3 region, mice were given saline or Rb1. Control shRNA and α-synuclein shRNA mice were intraperitoneally injected with vehicle (saline) from day 1 to day 14. α-Synuclein shRNA+Rb1 mice were intraperitoneally injected with Rb1 from day 1 to day 14. One day after the last Rb1/saline injection (day 15), behavioral tests and electrophysiological recording were performed. (**C**–**F**) Morris water maze tests were conducted after treatment with α-synuclein shRNA and Rb1. Mice were analyzed for (C) the escape latency during a 5-day training course. In the probe tests, mice were analyzed (D) for the escape latency, (E) the time spent in the target zone, and (F) the target crossing to reach the target platform from the entrance. n = 12 per group. (**G**) LTP at the SC-CA1 synapses was recorded in mice treated with α-synuclein shRNA or α-synuclein shRNA + Rb1. The middle image shows representative traces of fEPSP recordings of responses before and 50 min after high-frequency stimulation (HFS; arrow). (**H**) Quantitative analysis of LTP data in (G). The level of fEPSP potentiation was determined at a mean of 0–3 min and 50–60 min after high-frequency stimulation. n = 6. Results are expressed as the mean ± SEM. ^**^p < 0.01, ^*^p < 0.05 vs. control shRNA group. Statistical significance was determined by one-way ANOVA and Bonferroni tests as *post hoc* comparisons.

We found that α-synuclein knockdown decreased PSD-95 expression, and Rb1 only affected α-synuclein and PSD-95 expression in the primary hippocampal neurons in MPTP-treated mice. We then hypothesized that Rb1’s prevention of memory deficits in MPTP-treated mice may involve regulation of the α-synuclein/PSD-95 pathway. To confirm our hypothesis, we generated α-synuclein shRNA, and we provide the detailed vector map in Supplementary Figure 5A. We also confirmed the shRNA interference efficiency in HEK-293T cells (Supplementary Figure 5B). To study the effects of α-synuclein shRNA on the α-synuclein/PSD-95 pathway we performed Western blots, which suggested that α-synuclein shRNA also decreased PSD-95 expression (α-synuclein: F _3, 20_ = 13.750, *P* = 0.002, post-hoc *P* = 0.152, 0.001 and 0.001 for 1, 3 and 5 μl α-synuclein shRNA, respectively; PSD-95: F _3, 20_ = 11.306, *P* = 0.003, post-hoc *P* = 0.550, 0.008 and 0.001 for 1, 3 and 5 μl α-synuclein shRNA, respectively; Figure 5A), consistent with the *in vitro* findings. Here, we chose to deliver 3 µl α-synuclein shRNA into both hippocampal CA3 regions. LV-α-synuclein shRNA or control shRNA virus was stereotaxically injected, and seven days after virus injection, we intraperitoneally injected Rb1 (40 mg/kg) or saline for another 14 days. This timeline is shown in Figure 5B.

We first examined the effect of α-synuclein shRNA on spatial learning and memory using the Morris water maze test. Mice injected with α-synuclein shRNA showed impaired learning response with increased escape latency during the five-day training phase while Rb1 treatment (40 mg/kg) prolonged the escape latency to the target (Figure 5C). Furthermore, in the probe trial test, in which the hidden platform was removed on Day 6, both α-synuclein shRNA mice and α-synuclein shRNA mice treated with Rb1 spent considerably more time to the target compared with controls (F _2, 33_ = 6.595, *P* = 0.005, post-hoc *P* = 0.045 for α-synuclein shRNA, and post-hoc *P* = 0.001 for α-synuclein shRNA+Rb1; Figure 5D). α-Synuclein shRNA mice treated with Rb1 spent less time in the target quadrant (F _2, 33_ = 20.881, *P* < 0.001, post-hoc *P* = 0.274 for α-synuclein shRNA, and post-hoc *P* = 0.001 for α-synuclein shRNA+Rb1; Figure 5E), while both α-synuclein shRNA and α-synuclein shRNA mice treated with Rb1 had fewer target crossings compared with controls (F _2, 33_ = 6.328, *P* = 0.005, post-hoc *P* = 0.046 for α-synuclein shRNA, and post-hoc *P* = 0.001 for α-synuclein shRNA+Rb1; Figure 5F). Additionally, α-synuclein shRNA or α-synuclein shRNA mice treated with Rb1 showed no difference in swim speed in the five-day training phase or in the probe trial test (Supplementary Figure 6). We also examined the effects of α-synuclein shRNA on the locomotor activity, exploration, and emotional reactivity of mice using the open field test, T-maze, and EPM test. Consistent with previous reports [33, 47], α-synuclein knockdown in the hippocampal CA3 region did not affect locomotor activity, exploration, or anxiety-like behavior compared with control mice (Supplementary Figure 7 and 8). These results suggest that Rb1’s regulation of spatial learning and memory may involve α-synuclein.

We then recorded the fEPSPs in the hippocampal CA1 stratum radiatum. Both the post-tetanic potentiation and LTP at SC-CA1 synapses were reduced in hippocampal slices from the α-synuclein shRNA and α-synuclein shRNA mice treated with Rb1. Moreover, α-synuclein shRNA decreased the LTP amplitude at 0–3 min and 50–60 min after LTP induction, and Rb1 indicated no effect on the LTP in mice injected with α-synuclein shRNA (F _2, 15_ = 71.865, *P* < 0.001, post-hoc *P* = 0.048 for α-synuclein shRNA and post-hoc *P* = 0.027 for α-synuclein shRNA+Rb1 at 0-3 min; F _2, 15_ = 183.463, *P* < 0.001, post-hoc *P* = 0.003 for α-synuclein shRNA and post-hoc *P* = 0.008 for α-synuclein shRNA+Rb1 at 50-60 min; Figure 5G and 5H). Since α-synuclein is mainly localized at excitatory presynapses and co-expressed with vGluT1, a lack of α-synuclein impairs the mobilization of glutamate from the reserve pool [48], we also examined vGluT1 expression in the α-synuclein knockdown mice. Here we found that α-synuclein knockdown decreased vGluT1 expression in the hippocampus, and Rb1 could not rescue vGluT1 expression in the α-synuclein knockdown mice (F _2, 15_ = 82.343, *P* < 0.001, post-hoc *P* < 0.001 for α-synuclein shRNA and α-synuclein shRNA+Rb1; Supplementary Figure 9). These findings suggest that Rb1’s regulation of memory function and hippocampal synaptic plasticity may be dependent on α-synuclein.

**Figure 6:**
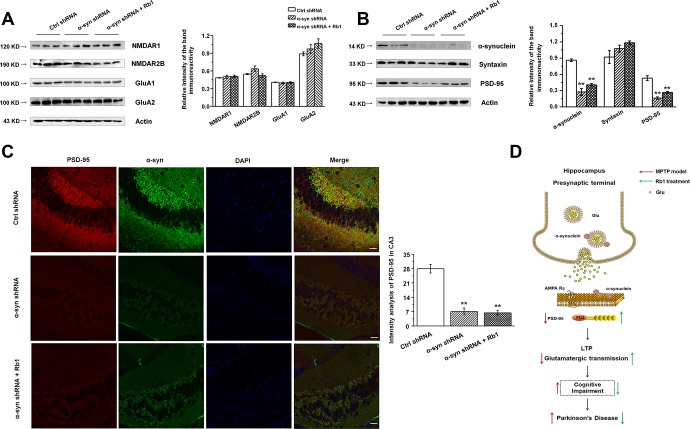
**Effect of CA3 α-synuclein knockdown on the glutamate receptors and synaptic expressions in the hippocampus.** (**A** and **B**) LV- α-synuclein shRNA or control shRNA virus was stereotaxically injected in the hippocampal CA3 region, and the effect of α-synuclein knockdown on the glutamate receptors and synaptic expressions in the hippocampus was determined by Western blotting. © Double staining of α-synuclein with PSD-95 in the hippocampal CA3 was shown. n = 10 per group. Scale bar, 80 μm. (**D**) Schematic model for Rb1-mediated improvement of cognitive deficits in the MPTP mouse model of PD. Western blotting results are from three of the nine mice in each group and are expressed as the mean ± SEM of three experiments. ^**^p < 0.01 vs. control shRNA group. Statistical significance was determined by one-way ANOVA and Bonferroni tests as *post hoc* comparisons.

**Figure 7:**
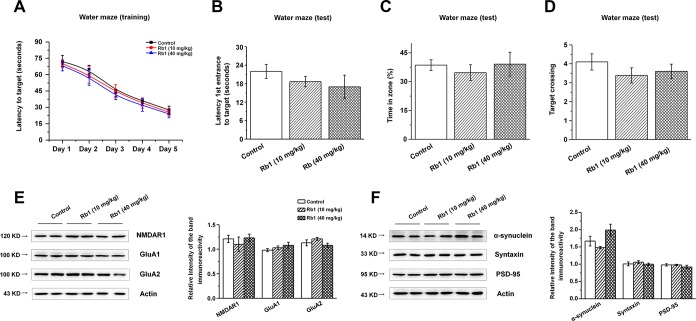
**Effect of Rb1 treatment on the glutamate receptors and synaptic expressions in the hippocampus in normal mice.** (**A**–**D**) Morris water maze tests were conducted after treatment with Rb1 (10 mg/kg or 40 mg/kg) in normal mice. Mice were analyzed for (A) the escape latency during a 5-day training course. In the probe tests, mice were analyzed (B) for the escape latency, (C) the time spent in the target zone, and (D) the target crossing to reach the target platform from the entrance. n = 8 per group. (**E** and **F**) Effects of Rb1 on the glutamate receptors and synaptic proteins expressions in the hippocampus in normal mice. Western blotting results are from two of the six mice in each group and are expressed as the mean ± SEM of three experiments. Statistical significance was determined by one-way ANOVA and Bonferroni tests as *post hoc* comparisons.

We then examined the expressions of glutamate receptors and synaptic proteins in the hippocampus, and we found that α-synuclein shRNA significantly decreased α-synuclein and PSD-95 expressions, while showing no obvious effects on the NMDA receptors (NMDAR1, NMDAR2B), AMPA receptors (GluA1, GluA2), or presynaptic protein (syntaxin) (Figure 6A and 6B). Furthermore, Rb1 did not prevent α-synuclein or PSD-95 expression in mice injected with synuclein shRNA (α-synuclein: F _2, 24_ = 11.334, *P* = 0.009, post-hoc *P* = 0.003 for α-synuclein shRNA, and post-hoc *P* = 0.006 for α-synuclein shRNA+Rb1; PSD-95: F _2, 24_ = 29.594, *P* = 0.001, post-hoc *P* < 0.001 for α-synuclein shRNA, and post-hoc *P* = 0.002 for α-synuclein shRNA+Rb1; Figure 6B). Immunofluorescence results also revealed that α-synuclein shRNA significantly decreased PSD-95 expression, while Rb1 did not prevent PSD-95 expression in the hippocampal CA3 region (F _2, 27_ = 14.698, *P* < 0.001, post-hoc *P* < 0.001 for α-synuclein shRNA and α-synuclein shRNA+Rb1, respectively; Figure 6C). The schematic model for how Rb1 improving cognitive impairment in MPTP mice model was shown in Figure 6D. Furthermore, we found that 10 or 40 mg/kg Rb1 only treated group showed no obvious effects on the spatial learning and memory performance in the Morris water maze test in the normal C57BL/6 mice (Figure 7A–7D). Rb1 treatment in normal mice (10 mg/kg or 40 mg/kg) showed no difference in swim speed in the five-day training phase or in the probe trial test as compared with control (Supplementary Figure 10). We also found that Rb1 showed no obvious effects on the glutamate receptors and synaptic protein expression in the hippocampus in the normal mice (Figure 7E and 7F), suggesting Rb1 may have no obvious effects on the cognitive function of the normal mice.

Hence, we conclude that Rb1’s regulation of memory function and hippocampal synaptic plasticity may involve the α-synuclein/PSD-95 pathway in the MPTP mice model of PD.

## DISCUSSION

Increasing evidence indicates that cognitive deficits are a significant non-motor symptom of PD. These cognitive-domain impairments occur early in PD, even prior to dopaminergic treatment [49], suggesting that α-synuclein pathology may be largely responsible for this process. Described as a significant pathologic phenotype in synucleinopathies, α-synuclein fibrils can also observed to reduce dendritic spine densities, the frequency, and amplitudes of spontaneous Ca^2+^ transients in hippocampal neurons and impair LTP via NMDA receptor activation [50, 51]. However, under physiological conditions, α-synuclein is localized at presynaptic terminals and is involved in the release of synaptic vesicles [27–29]. Moreover, α-synuclein knockout mice have been shown to have deficiencies in working and spatial memory tests [33], suggesting that α-synuclein also plays an important role in learning and memory. However, the normal function of hippocampal α-synuclein in the cognitive deficits in PD remains largely unclear. In this study, we demonstrated that Rb1 improves the spatial learning and memory deficits via upregulation of the trans-synaptic hippocampal α- synuclein/PSD-95 pathway in MPTP-treated mice.

Many animal models have been developed to study PD, including the classic administration of neurotoxins (such as 6-OHDA and MPTP). MPTP lesioning is a widely used PD animal model. Generally, MPTP in the brain is metabolized to 1-methyl-4-phenyl-2,3-dihydropyridinium by the enzyme monoamine oxidase B (MAO-B) within non-dopaminergic cells, and then converted to MPP^+^, an active, toxic compound of MPTP. MPP^+^ is taken into DA neurons via DA transporters, causing mitochondrial dysfunction and thus inducing DA neuron degeneration. Though MPTP administration is an established model for studying DA neuron degeneration, some groups also used MPTP to study its effects on the cognitive function in PD [52–54]. The mechanism may involve the MPTP decreasing synaptic synaptophysin, PSD-95, SNAP25 or synaptophysin, which may be due to alteration of presynaptic integrity medicated by striatal dopamine denervation caused by loss of dopaminergic neurons or connections [52, 55]. MPTP also affects basal synaptic transmission by modulation of presynaptic vesicle release via dopamine D2-like receptors [56]. The MPTP model has also been proposed to induce hippocampal memory deficits, involving the inactivation of the BDNF-TrkB (tyrosine kinase receptor B) pathway and reduced calcium/calmodulin-dependent protein kinase II activity [39, 41]. In addition, oxidative stress, mitochondrial dysfunction and inflammation also contribute to the effects of MPTP on the cognitive impairment [53, 57]. Thus, MPTP administration is suitable for elucidating the cognitive impairment associated issues. In this study, we present evidence indicating subacute MPTP intoxication of mice result in memory deficits, suggesting this model could be considered for the study of cognitive function in PD. Previously, we used the subacute MPTP mouse model to examine the role of glutamate transporter GLT-1 in regulating motor function, and reported that MPTP increased α-synuclein expression in the substantia nigra [35, 44, 58]. However, little is known regarding the effect of MPTP on hippocampal α-synuclein expression. Our findings indicate that MPTP induced decreased monomer rather than increased oligomer α-synuclein expression (Figure 3B), suggesting that MPTP did not induce α-synuclein aggregation, at least in the hippocampal CA3 region.

Previously, we and other groups have reported that MPTP injection can impair the LTP [39, 44], and here, for the first time, we report that MPTP also decreased the amplitudes and frequencies of AMPA receptor- mediated mEPSCs in CA3 pyramidal neurons. The decreased availability of membrane AMPA receptor subunit GluA1 receptor in the hippocampus may likely be responsible for the reduced amplitudes of mEPSCs. However, the unchanged presynaptic proteins, such as synapsin, synaptophysin, and syntaxin, may not explain why MPTP induced the decrease of mEPSC frequency. As α-synuclein is involved in presynaptic glutamate release, and the Schaffer collateral inputs from CA3 to CA1 is also glutamatergic we hypothesize that reduced α-synuclein expression in the CA3 region may be responsible for the MPTP-mediated decrease of LTP and mEPSCs. In this study, we found α-synuclein monomer rather than oligomer is decreased in the hippocampus (Figure 3), and other synaptic proteins remain unchanged, suggesting reduced α-synuclein may decrease presynaptic glutamate release, and the impaired glutamatergic transmission induces cognitive disorder in the MPTP mice model. To test this hypothesis, we injected α-synuclein shRNA into the CA3 region of hippocampus, and we mimicked some distinguishing features of memory deficiency. For the first time, we report that α-synuclein knockdown in the hippocampal CA3 region induces memory deficits and reduces LTP, suggesting that α-synuclein may mediate MPTP-induced memory deficits and impaired hippocampal synaptic plasticity. These results are consistent with findings observed in α-synuclein knockout mice [33]. However, we found that α-synuclein knockdown decreased PSD-95 expression, while α-synuclein knockdown did not affect the NMDA and AMPA receptors, suggesting α-synuclein knockdown may modulate the glutamatergic transmission partially in a glutamate receptors independent manner. Furthermore, we detected no significant differences in the open field, T-maze, or EPM test in mice with hippocampal CA3 α-synuclein knockdown (Supplementary Figure 7 and 8), consistent with previous findings from α-synuclein knockout mice studies [47, 59].

In the hippocampal synapse, α-synuclein expression was highly concentrated in the granular and polymorphic layers of the DG and CA3 fields [30]. We also found that MPTP induced neuronal death in these areas (Figure 2A–2D). Because MPTP is metabolized to MPP^+^, and MPP^+^ is taken up by dopaminergic neurons, so MPTP induces significant dopamine depletion in the substantia nigra and striatum. Since midbrain dopamine neurons can bidirectionally regulate CA3-CA1 synaptic drive, and the progression of DAergic cell death correlates with impairments in CA1 synaptic plasticity and memory performance [60, 61], the hippocampal neuron death and cognitive impairment in this study may be also due to the dopamine depletion and the dysfunctional neural circuit between midbrain and hippocampus. Thus, in this study, we were unable to conclusively identify whether MPTP-mediated neuronal death involves regulation of α-synuclein.

Ginseng, the root of *Panax ginseng* C.A. Meyer (Araliaceae), is a widely used herbal medicine in treating Parkinson’s disease and Alzheimer’s disease in the Far East. Ginsenoside Rb1 is the main active ingredient of *Panax ginseng*, it has been demonstrated to protect DA neurons, SH-SY5Y cells, and PC12 cells from neurotoxicity, and it is also known to improve learning and memory in hippocampus-dependent tasks [34, 37, 38, 62, 63]. We also revealed that Rb1 suppresses glutamate excitotoxicity via increasing glutamate transporter GLT-1 expression and function through nuclear translocation of nuclear factor-kappa B (NF-κB) and that Rb1 can improve the motor deficits in the MPTP mouse model [35]. It is worth mentioning that Rb1 was reported to suppress the fibrillation and toxicity of α-synuclein and inhibited α-synuclein polymerization *in vitro* [64]. Additionally, our findings indicates that Rb1 can decrease α-synuclein expression in the substantia nigra [35]. However, the question of whether Rb1 is capable of improving cognitive impairment in PD and is capable of improving hippocampal α-synuclein expression remains undetermined. We propose that Rb1 is capable of preventing memory deficits, increasing LTP and glutamatergic transmission in CA3, as well as preventing hippocampal neuron loss in MPTP-treated mice. Furthermore, Rb1 also prevents α-synuclein and PSD-95 expression in the hippocampus. As stated previously, knockdown of α-synuclein induces memory deficits leads to decreased LTP and PSD-95 expression. We conclude that Rb1 is involved in the protection of memory function, and upregulation of LTP may be involved in the modulation of α-synuclein/PSD-95 pathway in MPTP-lesioned mice.

It is accepted that the formation and maintenance of LTP involves the mossy fiber-CA3 and CA3-Schaffer collateral-CA1 pathways, and the neurotransmitter glutamate is responsible for these pathways. As we mentioned previously, Rb1 did not adversely affect AMPA receptors, NMDA receptors, or presynaptic proteins, which involve presynaptic glutamate release and postsynaptic effects in MPTP-lesioned mice. Moreover, Rb1 showed no obvious effect on the expression of these proteins in primary hippocampal neurons *in vitro*. We then raised the question of how Rb1 increases the LTP and CA3 glutamatergic transmission in MPTP-lesioned mice. As α-synuclein is involved in regulating presynaptic glutamate release, Rb1 may restore the glutamate in the CA3-Schaffer collateral-CA1 pathway via upregulation of CA3 α-synuclein expression, thereby prolonging the longevity of LTP. Moreover, Rb1’s promotion of mossy fiber activity may also be beneficial towards the maintenance of LTP in MPTP-lesioned mice. Thus, α-synuclein is required for Rb1’s protection of memory function and synaptic plasticity in a PD animal model.

How does monomeric α-synuclein affect postsynaptic PSD-95, and how is it involved in regulating glutamatergic transmission? As mentioned above, α-synuclein is colocalized with presynaptic vGluT1, and modulates the glutamate release to some extent. In this study, we also found that vGluT1 expression was downregulated in the hippocampus of α-synuclein knockdown mice and Rb1 could not recuse vGluT1 expression. Together with our electrophysiological results, we conclude that α-synuclein plays an important role in the glutamatergic transmission (especially involves glutamate release), and this also contributes to the neuroprotective effects of Rb1 in the MPTP mice model of PD. Referring to how α-synuclein affect postsynaptic PSD-95, findings from Emanuele et al. indicated that extracellular human monomeric α-synuclein treatment induces fragmentation of lipid rafts, increases calcium entry, and leads to acute mobilization of synaptic vesicles and neurotransmitter release. It is reported that α-synuclein could bind phospholipidic membranes, interacting with specific microdomains, the lipid rafts [65], and PSD-95 is a lipid raft-anchored synaptic protein. Emanuele et al. also found that monomeric α-synuclein induces an acute increase in glutamatergic transmission at the postsynaptic terminal, with increased density of PSD-95 puncta [66], and this is also an evidence support that α-synuclein may affect PSD-95 via lipid raft. Thus, one reason may be as α-synuclein is a prion-like protein, endogenous α-synuclein released from the presynaptic cleft may likely bind the postsynaptic lipid rafts, disrupting the PSD-95 complex, and thereby result in a significant increase in the density of PSD-95. The other reason may be due to α-synuclein involves glutamate release, reduced α-synuclein may affect the glutamatergic transmission, as we reported the mEPSC results in this work (Figure 1G–1I). Previously, Ferreira et al. revealed that α-synuclein interacts with PrP^C^ to induce cognitive impairment via regulating mGluR5 and NMDAR2B [67]. Though we did not find the changed expression of NMDAR2B, as PSD-95 is a main excitatory postsynaptic protein, so α-synuclein may affect the PSD-95 expression via mGluR5 or other signaling cascades. Further research needs to explore the mechanism underlying α-synuclein effects on PSD-95. Recently, Yamada and colleagues [68] provided evidence that the physiological release of endogenous α-synuclein highly depends on intrinsic neuronal activity. This elevation of neuronal activity can rapidly increase α-synuclein release. In this study, MPTP induced hippocampal neuron death in DG and CA3 (Figure 2). We conclude that while neuronal death may decrease α-synuclein release, Rb1 protects hippocampal neurons from MPTP toxicity and successively increases the α-synuclein release. This may explain why Rb1 increases the expression of α-synuclein rather than other presynaptic proteins.

While we can conclude the monomeric α-synuclein is reduced in the hippocampal CA3 region in the MPTP mouse model, the question of whether the hippocampal monomeric α-synuclein also protects memory function in the early phases of PD still remains to be elucidated. Previously, Ardah et al. [64] reported that Rb1 can suppress the fibrillation and toxicity of α-synuclein *in vitro*, while the data from our study indicates that Rb1 is capable of decreasing the α-synuclein expression in the substantia nigra [35]. In this study, we found that Rb1 can increase α-synuclein expression in the hippocampus *in vivo* and *in vitro*. We think these two findings may be not contradictory—Rb1 may exert dual effects on α-synuclein in the different stages of PD pathogenesis. Although we found that Rb1 changed α-synuclein expression in the primary cultured hippocampal neuron *in vitro* and in the hippocampus of PD animal model, we did not find Rb1 treatment changed the cognitive performance in the normal mice, and we also did not find Rb1 treatment changed the hippocampal α-synuclein expression in the normal mice. We conclude that Rb1 mainly improves the cognitive impairment in the PD animal model, and Rb1-inducing different *in vitro* and *in vivo* α-synuclein expression pattern maybe due to the pharmacological metabolism of Rb1. Moreover, in this study, Rb1 treatment was started three days before MPTP treatment, and it underwent the whole MPTP lesion process. Thus, the model we used in this work is actually a neuroprotective one, and to explore this concept, further research is necessary to examine the pharmacological effects of Rb1 in PD models.

Overall, we provide evidence that Rb1 prevents memory deficits and impaired LTP and glutamatergic transmission in MPTP-treated mice. We found that Rb1 increases PSD-95 expression in an α-synuclein-dependent manner both *in vivo* and *in vitro*. Hence, we conclude that Rb1’s protection of memory function may involve regulation of the α-synuclein/PSD-95 pathway in MPTP-lesioned mice (as shown in Figure 6D). Our study indicates that Rb1 has potential as a therapeutic agent for PD patients with memory impairment.

## MATERIALS AND METHODS

### Reagents

MPTP (1-methyl-4-phenyl-1,2,3,6-tetrahydro pyridine) was purchased from Sigma-Aldrich (St. Louis, MO, USA). Ginsenoside Rb1 was purchased from MUST Biotechnology (Chengdu, China). Anti-MAP-2 (microtubule-associated protein 2), NMDAR1, and PSD-95 were purchased from Cell Signaling Technology (Danvers, MA, USA). Anti-NMDAR2B, GluA1, GluA2, α-synuclein, syntaxin, synaptotagmin, vGluT1, and integrin antibodies were purchased from Santa Cruz Biotechnology (Santa Cruz, CA, USA). Anti-GLT-1 and anti-GLAST antibodies were purchased from Proteintech (Rosemont, IL, USA). Anti-synapsin was purchased from Millipore (Billerica, MA, USA). Anti-synaptophysin were purchased from Sigma-Aldrich (St. Louis, MO, USA). Anti-actin antibody was purchased from Beyotime (Shanghai, China). Anti-Alexa Fluor 488-conjugated goat anti-mouse, anti-Alexa Fluor 594-conjugated goat anti-rabbit, horseradish peroxidase-conjugated goat anti-mouse, and rabbit antibodies were purchased from Boster (Wuhan, China). EZ-Link Sulfo-NHS-SS-Biotin was purchased from Thermo Fisher Scientific (Waltham, MA, USA; no. 21331). TUNEL (terminal deoxynucleotidyl transferase-mediated dUTP nick-end labeling) Staining Assay Kits were purchased from Beyotime (Shanghai, China). Trizol was purchased from Invitrogen (Carlsbad, CA, USA). PrimeScript RT Reagent Kits and SYBR Premix Ex Taq Kits were purchased from Takara (Otsu, Japan). A Timm staining kit was purchased from Gefan (Shanghai, China).

### Animals

Ten-week-old male C57BL/6 mice were obtained from SLAC Laboratory Animal Co., Ltd. (Shanghai, China). Three mice per cage had free access to food and water and were housed with a 12:12-h light/dark cycle with lights on from 06:00 to 18:00 and maintained at a constant temperature and humidity. They were allowed to adapt to the environment for at least 1 week before experiments. All experiments were conducted according to the National Institute of Health guidelines on the care and use of animals (NIH Publications No. 8023, revised 1978) and approved by the Institutional Animal Care and Use Committee of Guangzhou Medical University.

### Primary hippocampal neuron culture

Primary hippocampal neurons were derived from the hippocampu of C57BL/6 mice E16–18 pups. Briefly, the hippocampus was dissociated and the cells were collected following trypsinization. Dissociated cells were then plated on poly-l-lysine (0.33 mg/mL)-coated glass coverslips at a density of 5 × 10^5^ cells/cm^2^ in a 6-well plate and maintained in Neurobasal medium supplemented with B27, 1% penicillin/streptomycin, and ultraglutamine at 37°C under 5% CO_2_ air in an incubator. Glial growth was inhibited by adding cytosine β-d-arabinofuranoside (10 μM) 48 h after plating. Cells were grown for 14 days *in vitro* (DIV), ensuring half the medium was changed every 3 days. The presence hippocampal neurons were determined by immunostaining with anti-MAP-2 antibody expression.

### Drug treatment

The C57BL/6 mice were divided into four groups: saline control, MPTP group, low dose of Rb1 (10 mg/kg) plus MPTP, and high dose of Rb1 (40 mg/kg) plus MPTP. According to previous studies, the subchronic PD models for studying the memory function were generated by administration of MPTP intraperitoneally for 7 consecutive days at a dose of 30 mg/kg freebase (MPTP-HCl) in saline [39]. The vehicle for MPTP and Rb1 was saline. Rb1 was administered intraperitoneally for 14 consecutive days, starting 3 days before MPTP treatment, at a dose of 10 or 40 mg/kg. The time interval between MPTP and Rb1 injections was greater than 12 h. One day after the last Rb1/saline injection, electrophysiological recording and behavioral tests were performed at the same time.

To explore the effects of Rb1 on the cognitive function of normal mice, C57BL/6 mice were divided into three groups: control, low dose of Rb1 (10 mg/kg), and high dose of Rb1 (40 mg/kg). Rb1 was administered intraperitoneally for 14 consecutive days, and control was given the saline at the same time. One day after the last Rb1/saline injection, behavioral test was performed.

To explore the effects of Rb1 on α-synuclein and glutamate receptor expression, stock solutions of Rb1 were prepared in 0.01 M phosphate-buffered saline (PBS) and diluted to the appropriate concentrations using cell culture medium. Hippocampal neurons were treated with 1, 10, 50, and 100 µM concentration of Rb1 for 24 h, and the vehicle for Rb1 was PBS.

### α-Synuclein siRNA transfection in primary hippocampal neurons

Three small interfering RNA (siRNA) targeting α-synuclein sequences were designed as previously described: siRNA-1, 5-CAAAGAGCAAGUGACAAA-3′; siRNA-2, 5′-UGAGAAGACCAAAGAGCAA-3′; siRNA-3, 5′-GACAAAUGUUGGAGGAGCA-3 [69]. These three siRNAs were synthesized by RioBio (Guangzhou, China), and the negative control siRNA was also provided by RioBio. Transfection was performed according to our previous work [58]. In brief, siRNA stock solution was diluted to the working solution (100 nM) using riboFECT CP Buffer (RioBio, Guangzhou, China), and the diluted siRNA was incubated with riboFECT CP Reagent (RioBio) for 15 min at room temperature. The mixture was added into the culture medium and incubated with the cultured neurons. The α-synuclein mRNA expression was detected by qRT-PCR after transfection for 48 h. The α-synuclein protein expression was detected after a transfection period of 72 h.

### Stereotaxic injection of α-synuclein shRNA in hippocampus CA3

The interfering vector used in this study was pLKD-CMV-eGFP-U6-shRNA. The lentivirus vector (LV)-sh[α-synuclein] and LV-sh[control] were generated by ligating annealed oligonucleotides encoding sh α-synuclein (also see Supplementary Figure 5A) or a control sequence into the AgeI I/*EcoR* I site of the pLKD-CMV-eGFP-U6-shRNA vector. LV-sh[α-synuclein] was constructed to express shRNA targeting α-synuclein (GACAAATGTTGGAGGAGCA) from the U6 (RNA polymerase III) promoter to replace the former toxic ccdB sequence. LV-α-synuclein shRNA was constructed and the virus was packaged by OBio (Shanghai, China). Negative control shRNA (target sequence: TTCTCCGAACGTGTCACGT) was kindly gifted by OBio (Shanghai, China).

LV-α-synuclein shRNA or control shRNA virus was stereotaxically injected as described previously [58]. Briefly, mice were anesthetized and placed in a stereotaxic frame, and the lentivirus vector in 3.0 µl vol was delivered into both sides of the hippocampal CA3 at the target site (Bregma AP, −3.08 mm; ML, ±2.5 mm; DV, −3.75 mm). A Hamilton syringe was filled with LV-α-synuclein shRNA or control shRNA virus, and the needle was lowered into the tissue at a rate of 0.5 μL/min. The syringe was left in place for 5 min before being slowly withdrawn from the brain. Seven days after virus injection, we intraperitoneally injected Rb1 (40 mg/kg) or saline for another 14 days. One day after the last Rb1 injection, behavioral tests were performed and the mice in each group were sacrificed for the indicated experiments.

### Behavioral tests

### *Morris Water Maze (MWM) test*

The MWM test was performed as previously described [70]. The maze consisted of a pool (diameter: 120 cm) filled with water (22 ± 1°C) made opaque white with bright white food coloring. An invisible platform (10 cm^2^) that was submerged below the water surface (∼2 cm) was placed in the center of one of the four quadrants of the pool (NE, SE, SW, NW), and different images (circles, squares, and triangles) were hung on the pool walls. Mice were released in the water in one of the four quadrants randomly, the test was conducted daily over 5 consecutive days, and each mouse underwent two trials per day with an inter-trial interval of 30 min. Once the mouse located the platform, it was allowed to remain on it for ∼30 s. If the mouse failed to find the platform within 90 s, it would be gently guided to the platform by the trainer and remained there for 30 s. For probe trials, the platform was removed and the mice were allowed to swim freely for 90 s. Mice that had less motivation to swim were excluded from the experiment. The time of crossing through the original platform position, the time spent in the target quadrant, and the swimming speed were monitored by a camera. Images and swimming paths were stored in a computer and analyzed automatically using Smart 3.0 video tracking software (Panlab, Barcelona, Spain).

### *Open Field Test (OFT)*

The open field (OF) consisted of a square arena (50 cm × 50 cm) with a white floor and 40 cm high walls. The arena was brightly illuminated and had a central zone (25 cm × 25 cm) and a peripheral zone. Each mouse was gently placed in the center of the apparatus and observed for 5 min. The behavioral parameters (total distance traveled, time spent in the central and the peripheral zones, and total activity) were recorded with a video camera and analyzed with Smart 3.0 video tracking software (Panlab, Barcelona, Spain). After each trial, the apparatus was cleaned with 75% ethanol.

### *T-Maze*

The T-maze apparatus was constructed from gray plastic and consisted of three arms (50 cm × 10 cm, walls 15 cm high). To examine the exploration activity of each mouse within the novel arm, it was blocked with a plastic slide door (defined as the novel arm), and mice were allowed to enter the two open goal arms for 5 min. The blocking door was then removed after 30 min, and the mice were returned and allowed to enter either of the open arms freely for another 5 min. The time spent in each arm was calculated visually after video recording by Smart 3.0 video tracking software. The percentage of time spent in the novel arm was calculated as the ratio of time spent in the new arm/total time spent in the three arms for each group. Following each trial, the apparatus was cleaned with 75% ethanol.

### *Elevated Plus Maze (EPM) test*

The EPM apparatus contained two open arms (L 25 cm × W 5 cm) across from each other and two enclosed arms (L 25 cm × W 5 cm × H 15 cm) across from each other and had an open roof. The maze was set at 50 cm above the floor. Mice were placed in the center, and their behavior was recorded for 5 min with a camcorder located above the maze. The parameters (the percentage of entries into the open arms, closed arms, and central platform and the percentage of time spent in the open arms, closed arms, and central platform) were calculated visually after video recording by Smart 3.0 video tracking software. Following each trial, the apparatus was cleaned with 75% ethanol.

### *Motor function-associated behavioral tests*

In this study, pole-climbing test, grasping test and rotarod test were performed as described in our recent work and other study [35, 44, 58, 71].

### Tissue preparation

Mice in each group were euthanized using isoflurane and tissues were collected for further analysis utilizing various assays. (1) Western blotting assays: Mice were anesthetized and perfused transcardially with 0.9% saline to remove traces of blood. The hippocampal tissues were removed and stored at −80°C. (2) Morphological experiments (colocalized immunofluorescence, immunohistochemistry, and TUNEL staining): Mice were anesthetized and perfused transaortally with 0.9% saline followed by fixative (4% paraformaldehyde in 0.01 M PBS, pH 7.4). The fixed brains were removed, stored overnight at 4°C in postfix solution (4% paraformaldehyde in 0.01 M PBS, pH 7.4), and dehydrated in a gradient of 20–30% sucrose. The embedded brains were cut into sections of 15 μm with a freezing microtome (Leica, Germany) and subsequently stored at −80°C before use. (3) Electrophysiological recording assay: The electrophysiological experiments were performed after the last Rb1/saline injection. Mice were anesthetized with isoflurane, the whole brain was removed, and the brain slice preparation was conducted as follows.

### Detergent-insoluble α-synuclein extraction

Alpha-synuclein insoluble fractions were extracted as described previously [72]. Hippocampus samples were homogenized and lysed in Triton soluble buffer (1% Triton X-100, 0.5 mM EDTA, 1 mM leupeptin, 1 mM aprotinin, 1 mM benzamidine, and 10 mM PMSF in TBS) and rotated at 4°C for 1 h. Then, the lysates were centrifuged at 14,000 rpm for 10 min, and the supernatant was collected as Triton soluble fractions. Pellets were resuspended in SDS soluble buffer (1% Triton X-100, 2% SDS, 1% Na deoxycholate, 1% NP40, 0.5 mM EDTA, 1 mM leupeptin, 1 mM aprotinin, 1 mM benzamidine, and 10 mM PMSF in TBS) and subjected to sonication. The samples were loaded for Western blotting.

### Total protein extraction and cell-surface biotinylation

Total protein lysis extraction and cell-surface biotinylation were performed as described previously [44].

### Western blotting assay

Prepared samples were subjected to gel electrophoresis (12% SDS-PAGE) and probed using relevant antibodies. Peroxidase activity was examined by enhanced chemiluminescence (Millipore, MA, USA), and chemiluminescent immunoreactive complexes were collected using the Tanon imaging system (Shanghai, China). Protein levels were quantified using ImageJ software. Actin or integrin immunoreactivity was set as the control.

### Immunofluorescence assay

The immunofluorescence assay was performed as described previously [44, 58]. Primary hippocampal neurons grown in the confocal dish or the prepared brain slices were incubated with primary antibodies overnight at 4°C, rinsed with PBS, and incubated with Alexa Fluor 488-conjugated goat anti-mouse and Alexa Fluor 594-conjugated goat anti-rabbit IgG for 2 h at 37°C. DAPI was used to stain cell nuclei. Immunostaining was then examined using an Olympus FV1000-1X81 laser scanning confocal microscope (Shinjuku, Tokyo, Japan).

### Immunohistochemistry assay

The immunohistochemistry assay was performed as described previously [58]. Hippocampal OD of α-synuclein immunostaining in the CA3 area shown in Figure 3a was determined by the Image-Pro Plus 6.0 photogram analysis system (IPP 6.0, Media Cybernetics, Bethesda, MD, USA) and was used as an index of CA3 density of α-synuclein innervation.

### TUNEL staining

Apoptotic cells in the hippocampal DG and CA3 regions were stained by TUNEL assay. The procedure was performed according to our previous work [44]. Briefly, prepared brain tissue slices as described in 2.8 were fixed in 4% paraformaldehyde and rinsed with PBS. Then, the slices were permeabilized with 1.0% Triton X-100 for 5 min, blocked with 5% BSA for 30 min, and the fluorescein TUNEL reagent mixture was incubated for 60 min at 37 °C. DAPI was used to stain cell nuclei, and then the slices were viewed using an Olympus FV1000-1X81 laser scanning confocal microscope (Shinjuku, Tokyo, Japan).

### TIMM staining

Timm staining was used to identify the hippocampal mossy fiber in the DG and CA3, and the staining was performed according to the manufacturer’s instructions. Briefly, mice were anesthetized and perfused intracardially with 150 ml of normal saline, followed by addition of 100 ml of 0.1 M PBS containing 1% sodium, 100 ml of 4% paraformaldehyde, and 50 ml of 0.1 M PBS containing 1% sodium. Then the brains were removed, fixed in 4% paraformaldehyde for 24 h, transferred to 0.1 M PBS containing 30% sucrose for another 48 h, and cut into 30 μm coronal sections. The hippocampal sections were stained in the dark for 90 min in a solution containing 60 ml of 50% arabic gum, 10 ml of 2 M citrate buffer, 15 ml of 0.5 M hydroquinone, and 15 ml of 17% silver nitrate at 26°C. The glass slides were washed in de-ionized water and dehydrated with gradient ethanol between 50% and 100%. Hippocampal OD of Timm immunostaining in DG and CA3 area was determined by the Image-Pro Plus 6.0 photogram analysis system (IPP 6.0, Media Cybernetics, Bethesda, MD, USA).

### Quantitative Real-time (RT)-PCR

Quantitative RT-PCR was performed as described previously [44]. The following primers were used in this study: α-synuclein, (forward) 5′-GCCAAG GAGGGAGTTGTGGCTGC-3′ and (reverse) 5′- CTGTTGCCACACCATGCACCACTCC-3′; β-actin, (forward) 5′-CTACAATGAGCTGCGTGTGGC-3′ and (reverse) CAGGTCCAGACGCAGGATGGC. Results were obtained by using the 2^−ΔΔCT^ method as described previously [44]. Data are from three separate experiments, each of which was performed in triplicate.

### Brain slice preparation in electrophysiological experiments

The electrophysiological experiments were performed after the last Rb1/saline injection. As we described previously [35], mice were anesthetized with isoflurane and quickly decapitated. Brains were rapidly removed and immersed in ice-cold oxygenated (95% O_2_, 5% CO_2_) sucrose artificial cerebrospinal fluid (ACSF) containing (in mM) sucrose (120), NaCl (64), KCl (2.5), NaH_2_PO_4_ (1.25), NaHCO_3_ (26), glucose (10), MgSO_4_ (10), and CaCl_2_ (0.5). Slices (350 μm) were prepared using a vibratome (Leica VT1000 S, USA) and then incubated in normal ACSF containing (in mM) NaCl (126), KCl (2.5), NaH_2_PO_4_ (1.25), NaHCO_3_ (26), glucose (10), CaCl_2_ (2), and MgSO_4_ (2), with continuous bubbling with 95% O_2_ and 5% CO_2_ at 32°C for 30 min, and then incubated at room temperature. All experiments were performed within 1–8 h after slice preparation.

### Electrophysiological recording

Following incubation, slices were transferred to a recording chamber in which oxygenated ACSF was warmed to 32°C and superfused over the submerged slices at 2 ml/min. Field excitatory postsynaptic potentials (fEPSPs) were evoked in the CA1 stratum radiatum by stimulating the Schaffer collateral (SC)/commissural pathway with a two-concentrical bipolar stimulating electrode (25-mm pole separation; FHC, Inc.) and, current clamp recording was conducted using an Axon instrument with a MultiClamp 700B amplifier (Molecular Devices) and aCSF-filled glass pipettes. Test stimuli consisted of monophasic 100-μs pulses of constant currents (with intensity adjusted to produce 25% of the maximum response) at a frequency of 0.0167 Hz. The strength of synaptic transmission was determined by measuring the initial (10–60% rising phase) slope of fEPSPs. CA1 area LTP was induced by one train of 100-Hz stimuli with the same intensity as the test stimulus, and a cut was made between CA1 and CA3 in hippocampal slices to prevent the propagation of epileptiform activity. Whole-cell patch-clamp recordings were collected from the hippocampal CA3 pyramidal neurons, with mEPSCs recorded at a holding potential of −65 mV, and slices thickness are 350 μm. For mEPSCs recording, glass pipettes were filled with internal solution containing (in mM) K-gluconate (120), KCl (20), HEPES (10), MgCl_2_ (2), EGTA (0.1), sodium phosphocreatine (10), leupeptin (0.2), Mg-ATP (4), and Na-GTP (0.3), as well as pH 7.3 (290 mOsm). GABA_A_ receptors were pharmacologically blocked with 20 μM bicuculline, and tetrodotoxin (1 μM) was included in the perfusion solution for mEPSC recording. Neurons were allowed to equilibrate for at least 5 min before recording. Data were acquired with MultiClamp 700B (Molecular Devices, Sunnyvale, CA) at 10 kHz, filtered at 1 kHz, and stored for later analysis using pClamp software (Molecular Devices). Only recordings for which the access resistance changed <15% were retained for analysis. Mini events were analyzed with Mini Analysis software (Synaptosoft, Inc.).

### Statistics

Statistical analysis of the data was performed on SPSS 16.0 (SPSS Inc., Chicago, IL) using one-way analysis of variance (ANOVA) followed by the Bonferroni *post-hoc* test for multiple comparisons and the Student's *t*-test for comparisons between two groups. All data are expressed as the mean ± standard error of the mean (SEM), and the statistical significance level was set at *P* < 0.05.

## Supplementary Material

Supplementary Figures
