# Supplementary material for: Ginsenoside Rb1 prevents MPTP-induced changes in hippocampal memory via regulation of the α-synuclein/PSD-95 pathway
Source: Aging (Albany NY). 2019 Apr 4;11(7):1934–64. doi: 10.18632/aging.101884 (PMC6503885; doi:10.18632/aging.101884)
Supplement: Supplementary Figures [file aging-11-101884-s001.pdf]

SUPPLEMENTARY MATERIALS

A

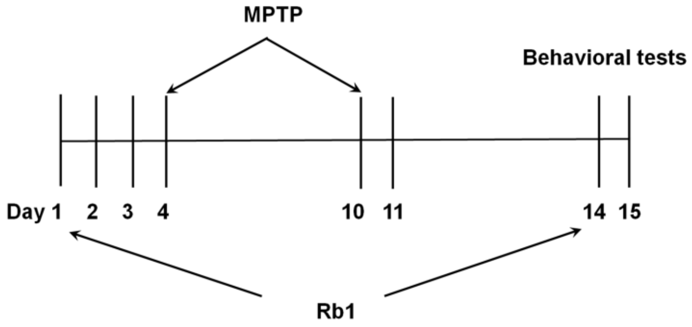

B

Groups	Assay 1 →	Assay 2	Number of mice in this group
Group 1	Behavioral tests of Rb1 treatment in MPTP mice （MWM, Pole-climbing test, Grasping test, and Rotarod test）	Immunofluorescence and Western Blotting	48
Group 2	Electrophysiological recording of Rb1 treatment in MPTP mice		52
Group 3	Behavioral tests of α-synuclein knockdown in normal mice （MWM)	Immunofluorescence and Western Blotting	36
Group 4	Electrophysiological recording of α-synuclein knockdown in normal mice		18
Group 5	Behavioral tests of Rb1 treatment in normal mice （MWM)	Western Blotting	24

Supplementary Figure 1. Time-line of the experiments. (**A**) Experimental timeline. Saline vehicle mice were intraperitoneally injected with vehicle (saline) once per day from day 1 to day 3 or from day 11 to day 14, and twice per day from day 4 to day 10. MPTP mice were intraperitoneally injected with vehicle (saline) once per day from day 1 to day 3 or from day 11 to day 14 and intraperitoneally injected with MPTP and saline from day 4 to day 10. Rb1 treatment mice were intraperitoneally injected with 10 or 40 mg/kg Rb1 once per day from day 1 to day 14 and intraperitoneally injected with MPTP from day 4 to day 10. The time interval between MPTP and Rb1 injections was more than 12 h (MPTP was given at 8:00 am and Rb1 was given at 8:00 pm). One day after the last Rb1/saline injection (day 15), behavioral tests and electrophysiological recording were performed. (**B**) We list the arrangements of different assays and the mice we used in each assay in the table.

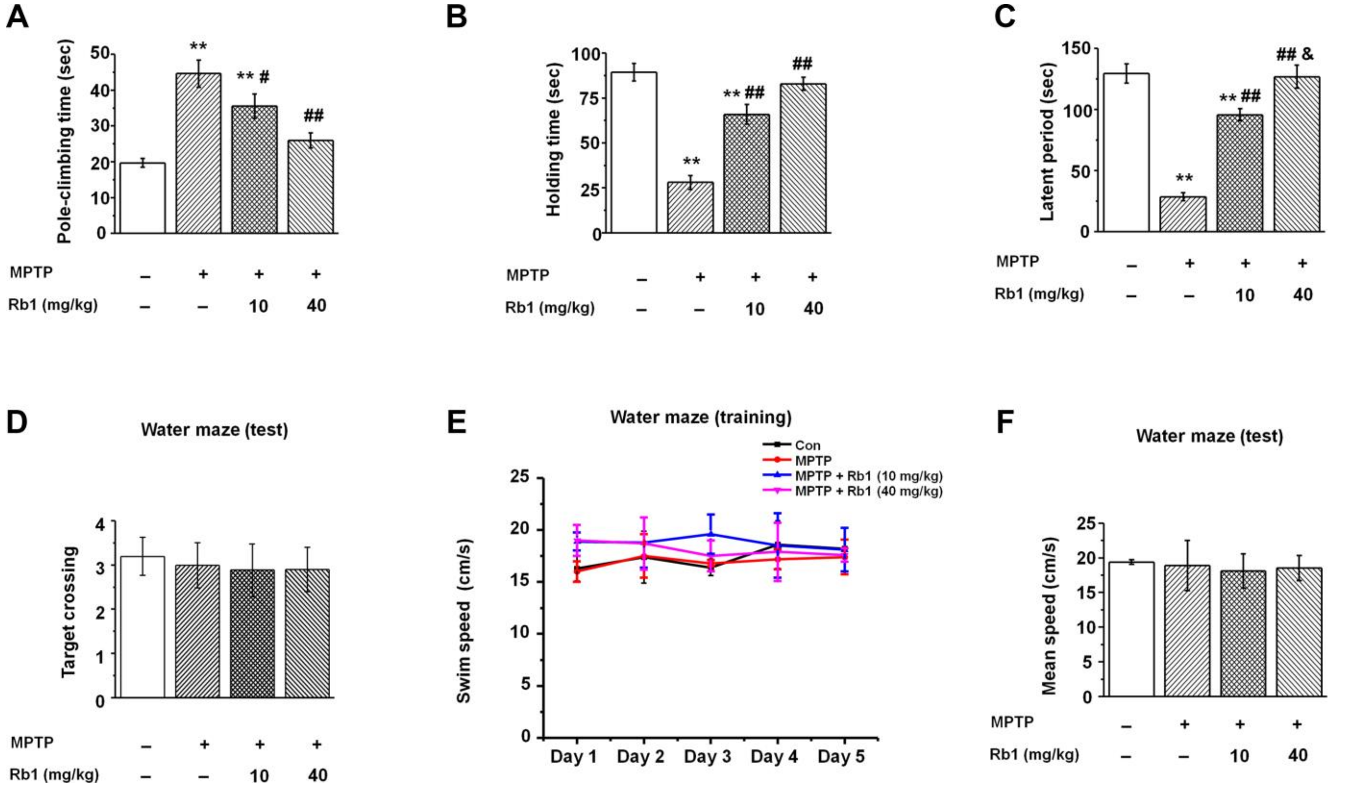

Supplementary Figure 2. Mice performance in the motor function-associated and Morris water maze test. (**A**–**C**) Behavioral test results of 10 and 40 mg/kg Rb1 on the motor deficits in Pole-climbing test (A), Grasping test (B) and Rotarod test (C) in MPTP-treated mice (n = 12 per group). (**D**) The frequency of target crossing to the target platform from the entrance in the Morris water maze tests. (**E**) Effect of Rb1 on the swim speed in the MPTP mice model in the five-day training phase. (**F**) Effect of Rb1 on the swim speed in the MPTP mice model in the probe trial test. n = 12 per group. * represents a significant difference as compared with the control group. $^{**}p < 0.01$. $^{\#}$ represents a significant difference as compared with the MPTP group. $^{\#\#}p < 0.01$, $^{\#}p < 0.05$. $^{\&}$ represents a significant difference as compared with the MPTP + Rb1 (10 mg/kg) group. $^{\&}p < 0.05$. Statistical significance was determined by one-way ANOVA and Bonferroni test as *post-hoc* comparisons.

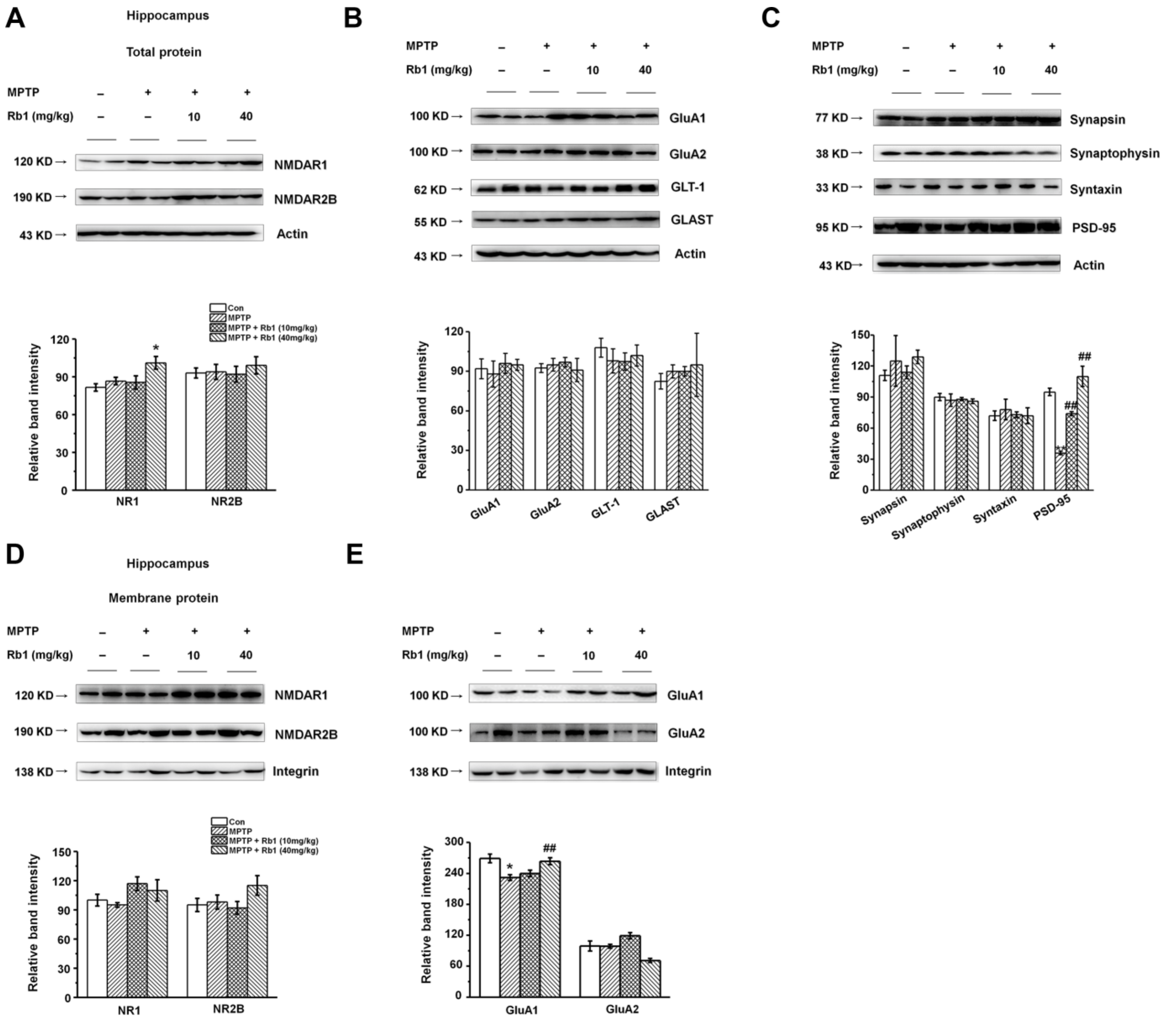

Supplementary Figure 3. Effect of Rb1 on the expression of glutamate receptors and synaptic proteins in MPTP-treated mice. (**A**–**C**) The effect of Rb1 on the glutamate receptors, glutamate transporters, and synaptic protein expression at the total protein level in the hippocampus in MPTP-treated mice was determined by Western blotting. (**D** and **E**) The effect of Rb1 on glutamate receptor expression at the membrane protein level in the hippocampus in MPTP-treated mice was determined by Western blotting. Western blotting results are from two of the six mice in each group and are expressed as the mean ± SEM of three experiments. $^{**}p < 0.01$, $^{*}p < 0.05$ vs. control group; $^{\#\#}p < 0.01$, $^{\#}p < 0.05$ vs. MPTP group. Statistical significance was determined by one-way ANOVA and Bonferroni tests as *post hoc* comparisons.

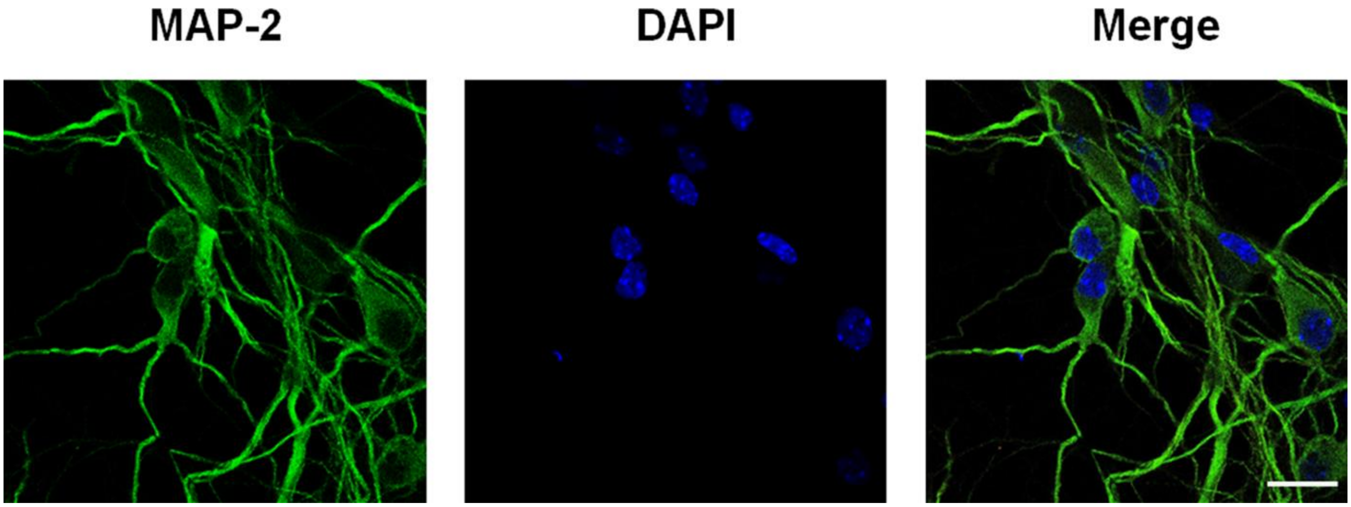

Supplementary Figure 4. Immunofluorescent staining of MAP-2 in primary cultured hippocampal neurons. Hippocampal neurons were cultured as described in Material and Methods, and the hippocampal neurons were stained by MAP-2. DAPI was used to stain the cellular nucleus. Scale bar, 30 μm.

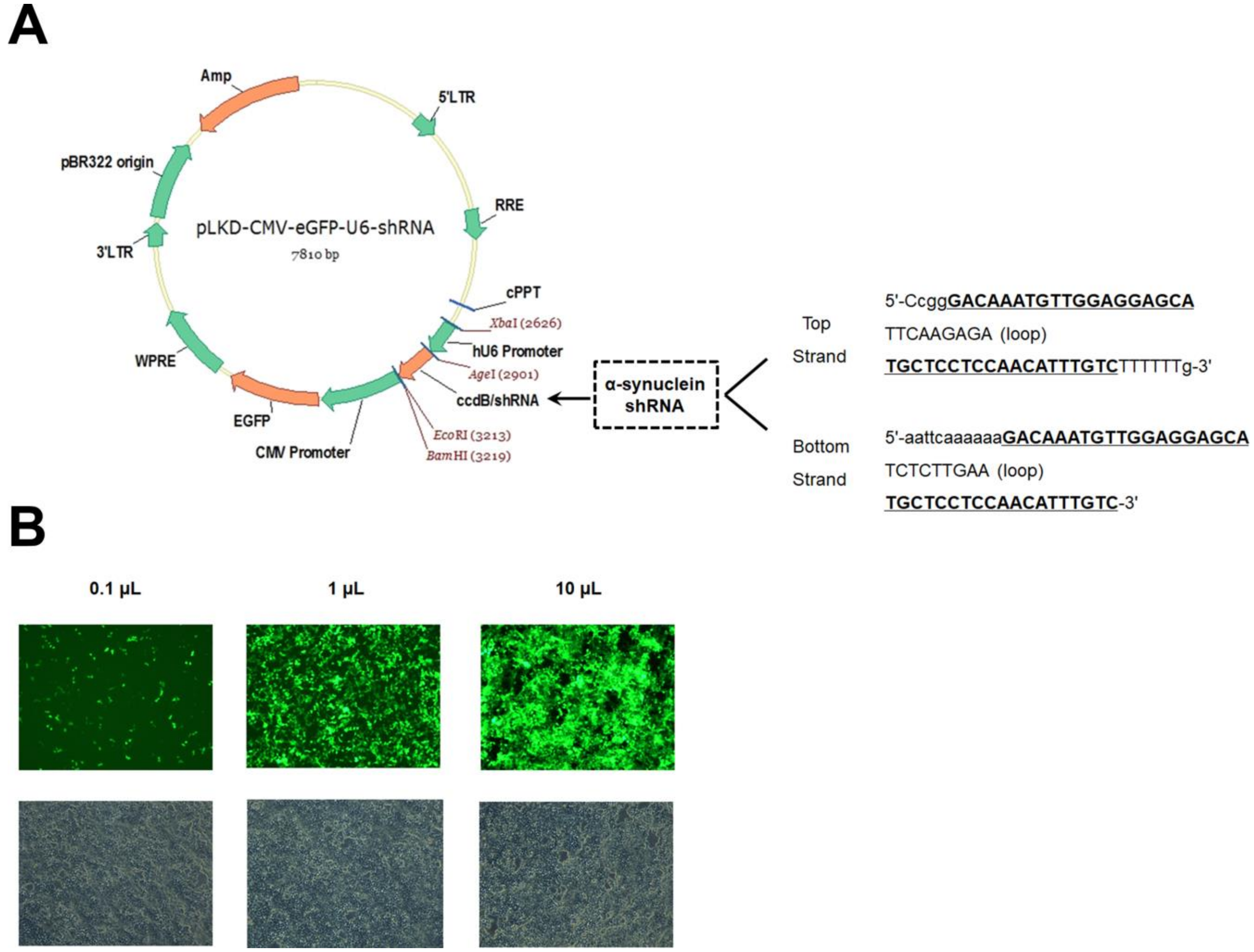

Supplementary Figure 5. Design of α-synuclein shRNA. (**A**) Design of an α-synuclein shRNA lentivirus vector (LV). The interfering vector used was pLKD-CMV-eGFP-U6-shRNA, and lentivirus vector (LV)-sh[α-synuclein] and LV-sh[control] were generated by ligating annealed oligonucleotides encoding sh α-synuclein or a control sequence into the AgeI I/*EcoR* I site of the pLKD-CMV-eGFP-U6-shRNA vector. LV-sh[α-synuclein] was constructed to express shRNA targeting α-synuclein (GACAAATGTTGGAGGAGCA) from the U6 (RNA polymerase III) promoter to replace the former toxic ccdB sequence. (**B**) The infection efficiency of different titers of α-synuclein shRNA was examined in HEK-293T cell.

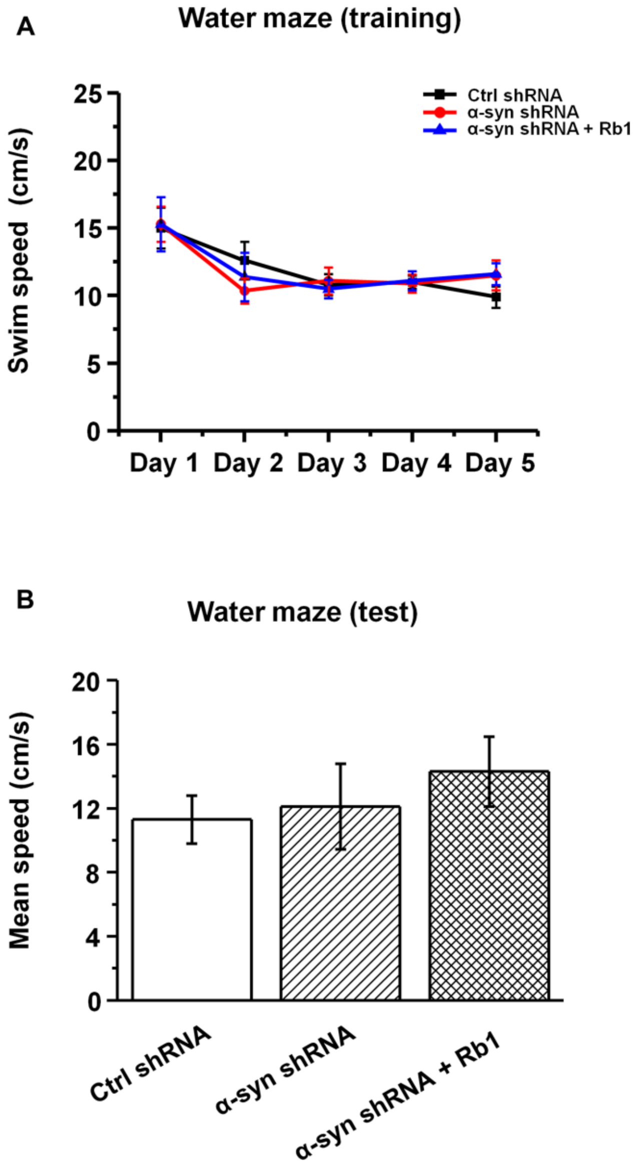

Supplementary Figure 6. Effect of α-synuclein shRNA on the swim speed in the Morris water maze test in the normal C57BL/6 mice. (**A** and **B**) LV-α-synuclein shRNA or control shRNA virus was stereotaxically injected in the hippocampal CA3 region, and the effect of α-synuclein shRNA or control shRNA on the swim speed in the Morris water maze test is shown. n = 12 per group. Results are expressed as the mean ± SEM. Statistical significance was determined by one-way ANOVA and Bonferroni tests as *post hoc* comparisons.

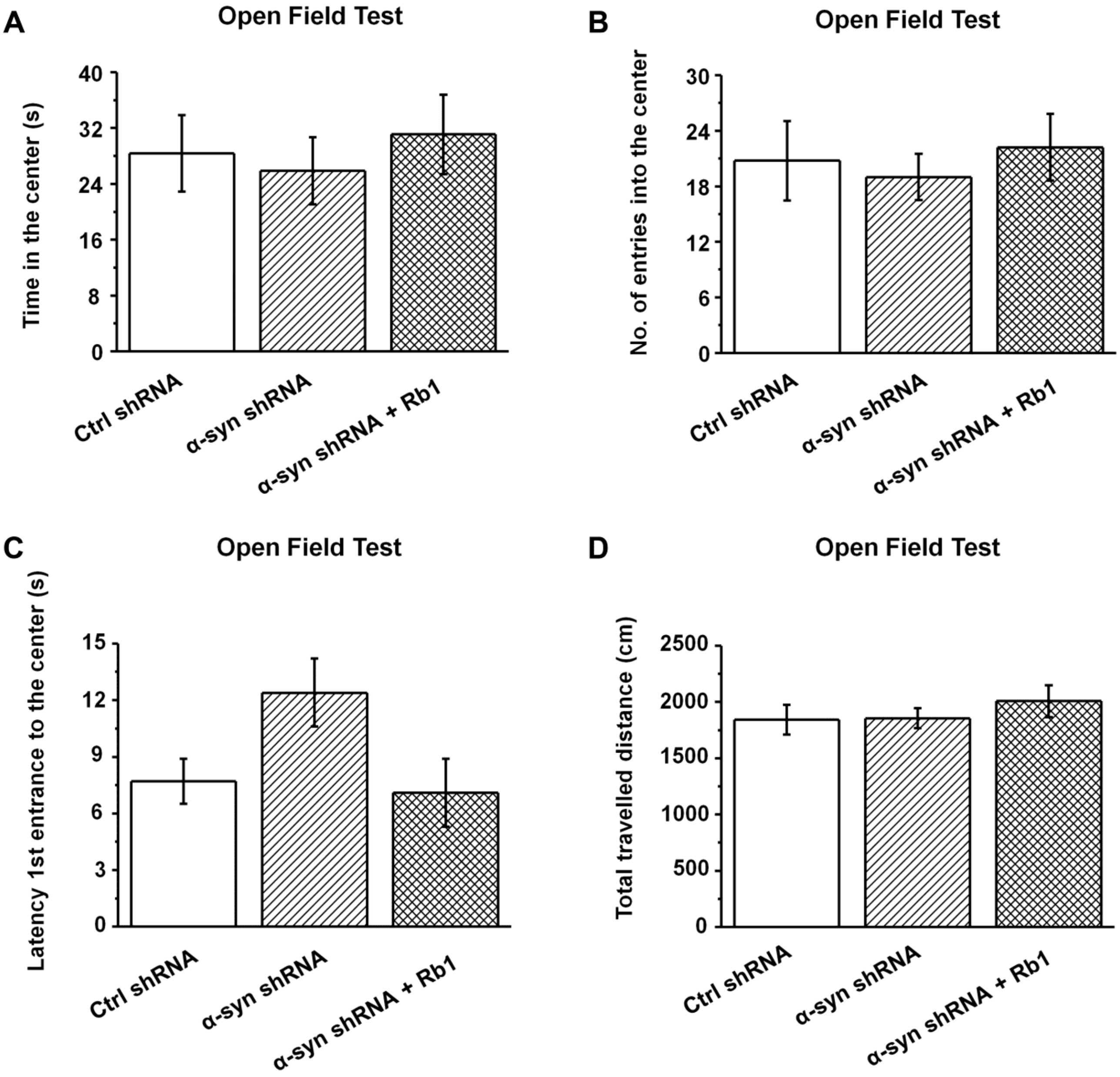

Supplementary Figure 7. α-synuclein shRNA did not affect the locomotor activity in the open field test. LV-α-synuclein shRNA or control shRNA virus was stereotaxically injected in the hippocampal CA3 region, and the open field test was performed. The results showed that α-synuclein shRNA did not affect the time in the center (**A**), number of entries into the center (**B**), latency to the center (**C**), or total travelled distance (**D**) in the open field test. n = 12 per group. Results are expressed as the mean ± SEM. Statistical significance was determined by one-way ANOVA and Bonferroni tests as *post hoc* comparisons.

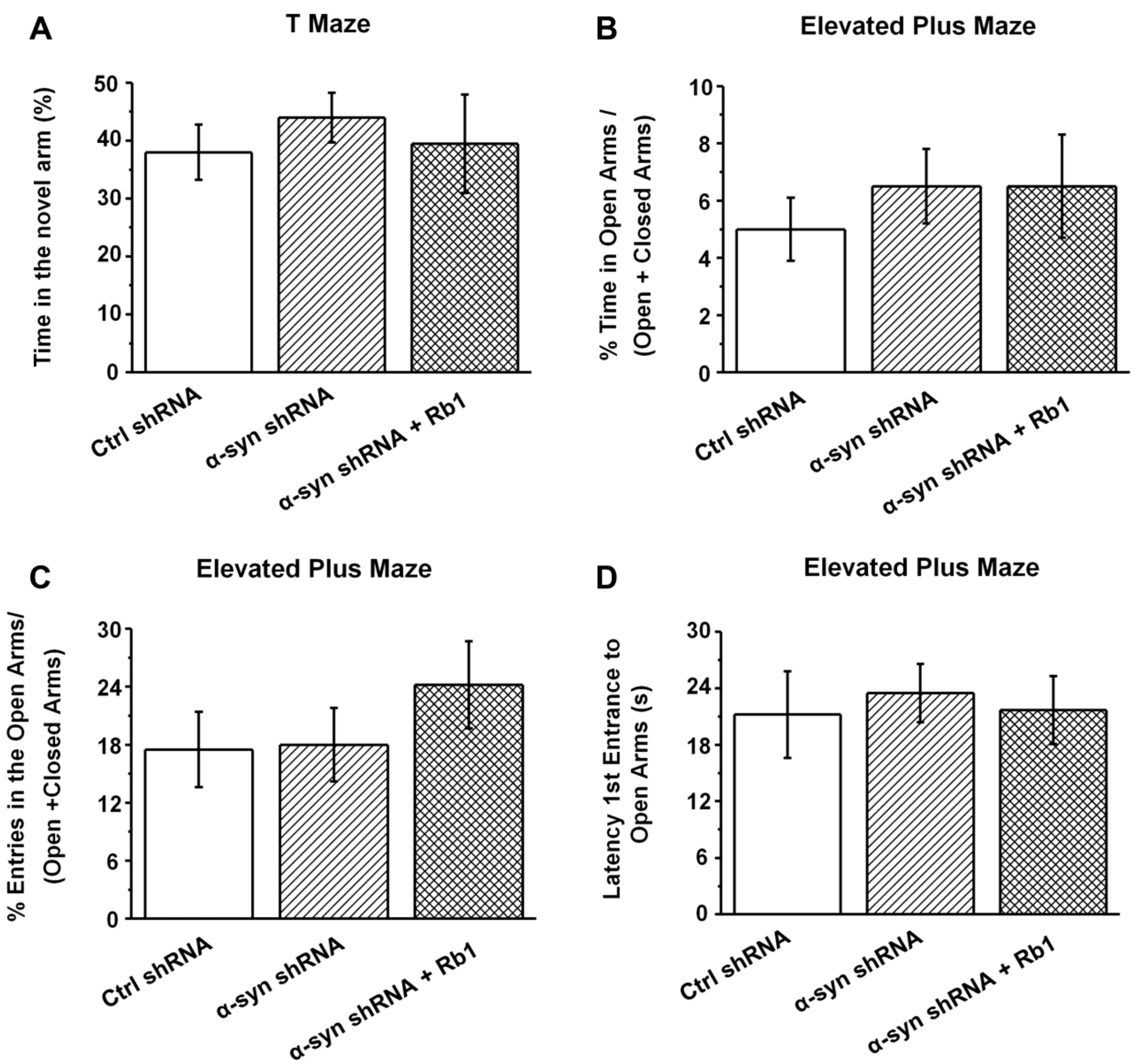

Supplementary Figure 8. α-Synuclein shRNA did not affect the emotional behavior in the EPM test. LV-α-synuclein shRNA or control shRNA virus was stereotaxically injected in the hippocampal CA3 region, and the open field test was performed. (**A**) α-Synuclein shRNA did not affect the performance of mice in the T-maze. (**B**–**D**) α-Synuclein shRNA did not affect the performance of mice in the EPM maze. The results showed that α-synuclein shRNA did not affect the time in open arms (B), entries in the open arms (C), or latency to the open arms (D). n = 12 per group. Results are expressed as the mean ± SEM. Statistical significance was determined by one-way ANOVA and Bonferroni tests as *post hoc* comparisons.

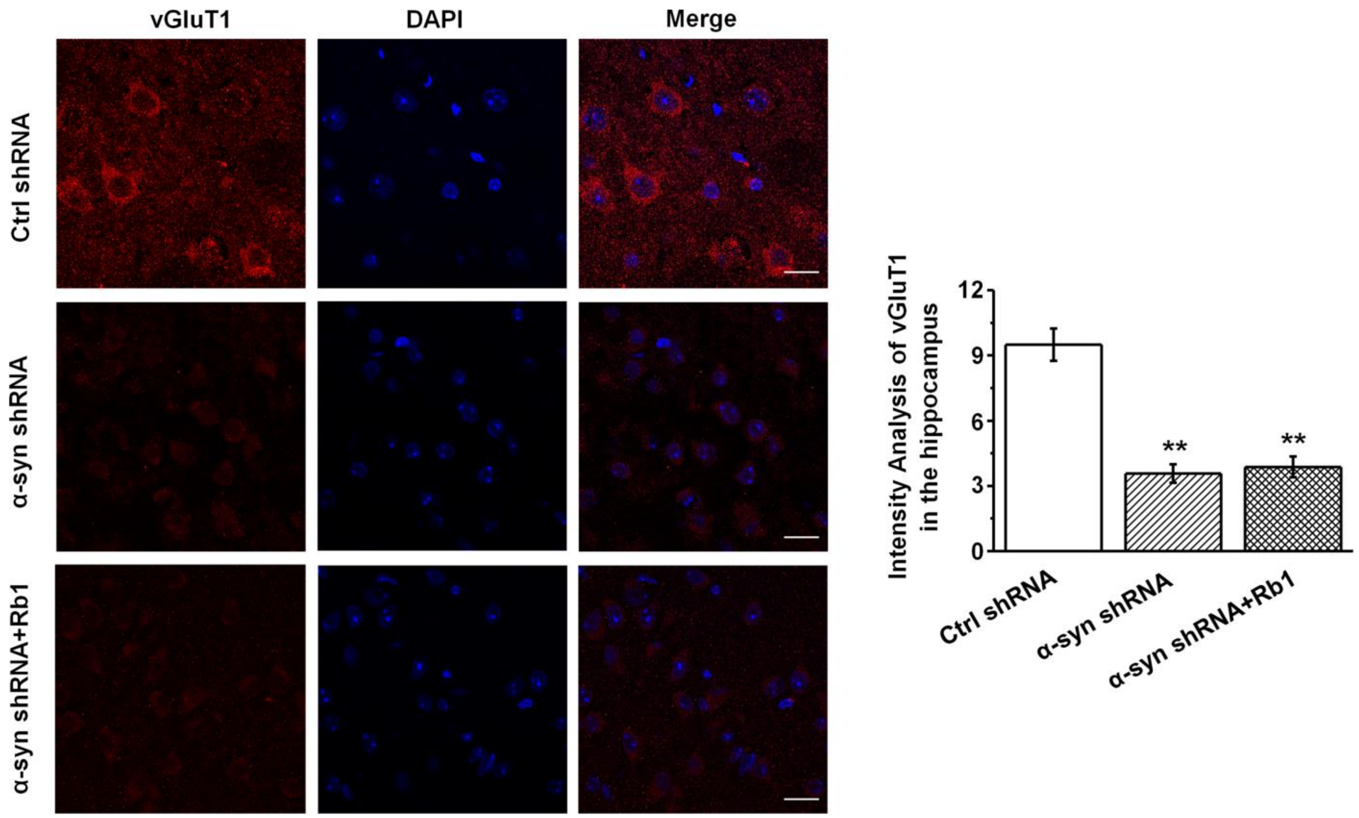

Supplementary Figure 9. Immunofluorescent staining of vGluT1 in the hippocampus in MPTP-treated mice. Mice were treated MPTP and Rb1 as described in Material and Methods, and the hippocampal slices were stained by vGluT1. DAPI was used to stain the cellular nucleus. Scale bar, 10 μm. * represents a significant difference as compared with the control group. $^{**}p < 0.01$.

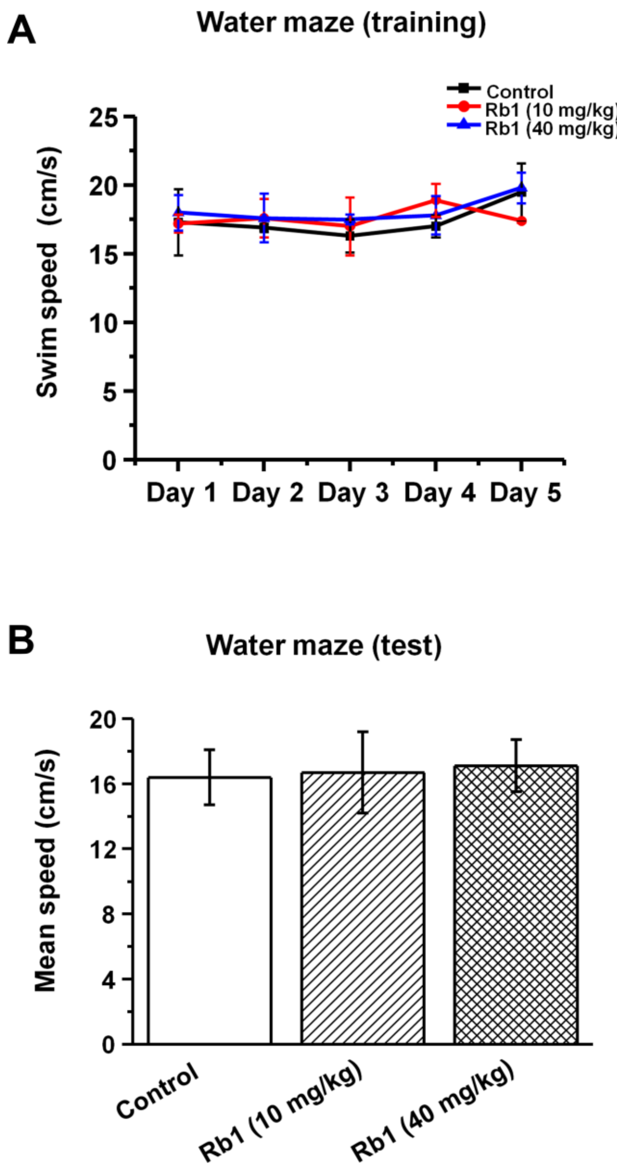

Supplementary Figure 10. Effect of Rb1 on the swim speed in the Morris water maze test in the normal C57BL/6 mice. (**A** and **B**) 10 mg/kg and 40 mg/kg Rb1 were treated in normal mice, and the effects of Rb1 treatment on the swim speed in the Morris water maze test were shown. n = 8 per group. Results are expressed as the mean ± SEM. Statistical significance was determined by one-way ANOVA and Bonferroni tests as *post hoc* comparisons.
